# Biomimetic PD-1-MSCs membrane-engineered nanoparticles for enhanced blood-brain barrier penetration and anti- glioma therapy

**DOI:** 10.3389/fimmu.2026.1906527

**Published:** 2026-08-18

**Authors:** Jie Li, Yuhao Gao, Wenbo Zhao, Huamin Zeng, Xuecen Zhao, Dan Deng, Shilin Chen

**Affiliations:** 1 Innovative Institute of Chinese Medicine and Pharmacy/Institute of Herbgenomics, Chengdu University of Traditional Chinese Medicine, Chengdu, China; 2 College of Pharmacy, Chengdu University of Traditional Chinese Medicine, Chengdu, China; 3 Department of Pathology, Chengdu Fifth People’s Hospital, Chengdu, China

**Keywords:** blood-brain barrier, elemene/cabazitaxel, glioma, immune remodeling, PD-1-functionalized biomimetic nanoparticles, tumor targeting

## Abstract

**Objective:**

Glioma is the most common and aggressive primary intracranial tumor. Its clinical management is substantially hindered by the blood–brain barrier (BBB) and the immunosuppressive tumor microenvironment (TME). This study aimed to construct PD-1-functionalized mesenchymal stem cell (MSC) membrane biomimetic nanoparticles co-loaded with elemene and cabazitaxel (PD-1-MSCs-ELE/CTX@BLIP) and investigate their capacity to penetrate the BBB, target gliomas, and their combined chemoimmunotherapeutic efficacy and related mechanisms.

**Methods:**

Genetic engineering was utilized to create PD-1-overexpressing MSCs, and the derived cell membranes were extracted to fabricate biomimetic nanoparticles. A series of characterizations was performed to ascertain the particle size, zeta potential, encapsulation efficiency, stability, and biosafety of the nanoparticles. An *in vitro* BBB model and glioma cell models were employed to evaluate BBB permeability, cellular uptake, cytotoxicity, cell cycle arrest, apoptosis, and inflammatory cytokine secretion. Subcutaneous and orthotopic glioma mouse models were created to investigate *in vivo* tumor targeting, antitumor activity, survival benefit, and systemic biosafety. Flow cytometry, immunofluorescence, enzyme-linked immunosorbent assay, and histopathological staining were employed to examine the regulatory impact of the nanosystem on the tumor immune microenvironment.

**Results:**

The synthesized PD-1-MSCs-ELE/CTX@BLIP exhibited uniform particle size, high drug encapsulation efficacy, and excellent storage stability and biocompatibility. Leveraging MSC biomimetic modification and the PD-1/PD-L1 axis, the nanoparticles exhibited significant BBB penetration ability and active targeting capability toward glioma cells (8.57-fold). *In vitro* studies revealed that the formulation significantly inhibited glioma cell proliferation and migration, induced cell apoptosis, and rejuvenated T cell exhaustion. *In vivo* findings revealed that PD-1-MSCs-ELE/CTX@BLIP exhibited significant accumulation in tumor tissues, remarkably suppressed tumor proliferation, and markedly prolonged the survival duration (57.52%) of orthotopic glioma-bearing mice. Mechanistically, it blocked the PD-1/PD-L1 immune checkpoint pathway, increased intratumoral CD8+ T cell infiltration, upregulated pro-inflammatory cytokine IFN-γ, and downregulated immunosuppressive factors IL-1β, thereby remodeling the immunosuppressive TME. Furthermore, major organs, blood, and liver/kidney function tests revealed no systemic toxicity.

**Conclusion:**

The PD-1-modified MSC membrane biomimetic nanoplatform integrates targeted chemotherapy and immune checkpoint inhibition. It efficiently crosses the BBB, specifically accumulates in glioma tissues, exerts potent antitumor effects, and improves the tumor immune microenvironment with favorable biosafety. This study provides a novel, promising biomimetic chemoimmunotherapeutic approach for precise glioma treatment.

## Graphical Abstract

**Figure:**
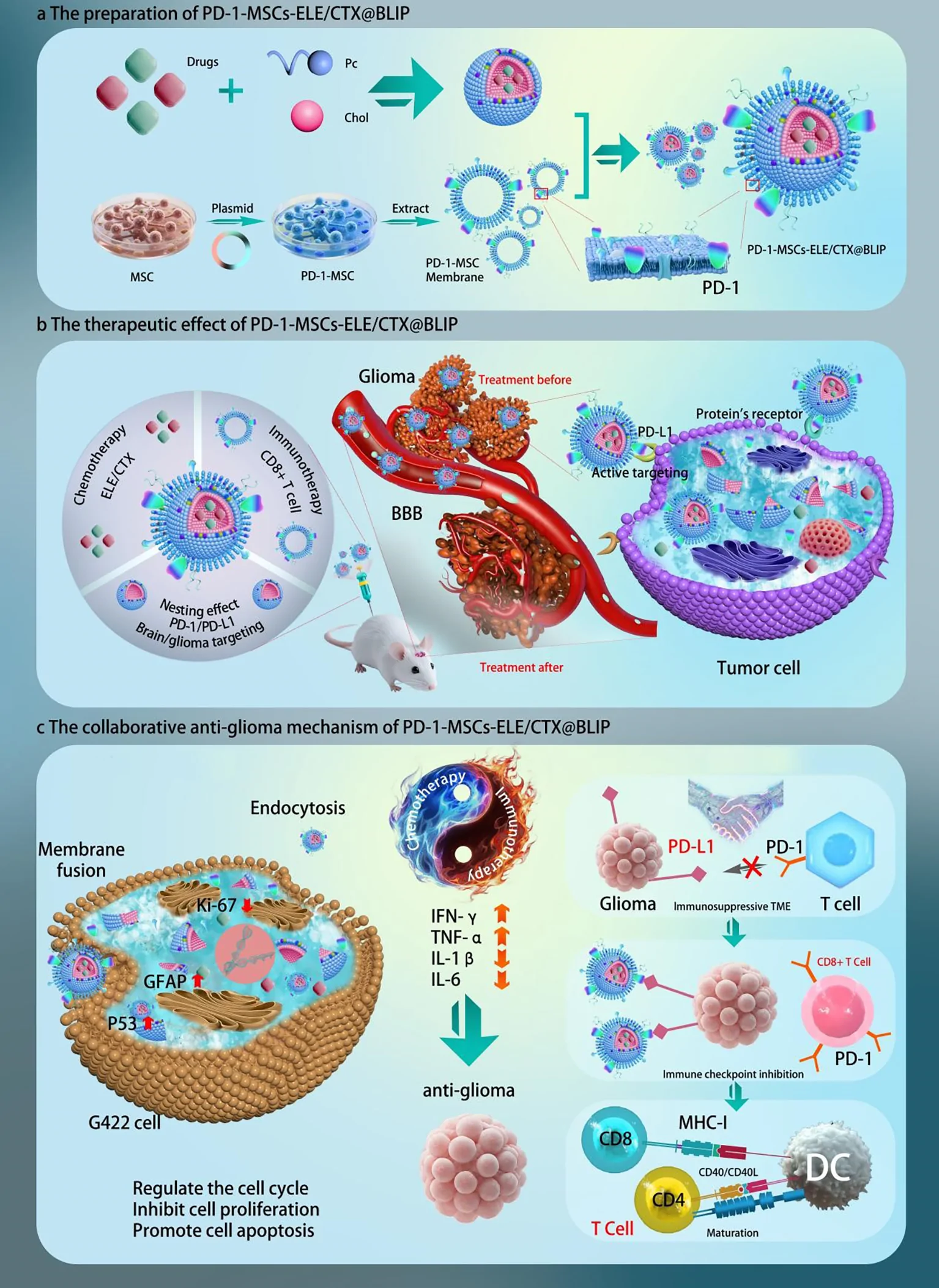
Schematic illustration of the fabrication, BBB penetration, active glioma targeting, and combined chemoimmunotherapeutic mechanism of PD-1-MSCs-ELE/CTX@BLIP. Phosphatidylcholine (Pc), cholesterol (Chol).

## 1 Introduction

Glioma is the most common and aggressive primary brain tumor, with a five-year survival rate of < 5% (1, 2). Its deep-seated location, a formidable blood-brain barrier (BBB), and an immunosuppressive tumor microenvironment (TME) render most systemic treatments ineffective (3, 4). Therefore, strategies that can simultaneously enhance BBB penetration and reinvigorate antitumor immunity are urgently needed (5, 6).

Nanodelivery systems represent a promising approach to overcoming the therapeutic challenges of glioma. Elemene (ELE) is a naturally occurring lipophilic active monomer derived from the traditional Chinese medicinal herb *Curcuma wenyujin* (温郁金), whose liposomal formulation has been extensively utilized in clinical tumor treatment because of its low systemic toxicity and mild immunoregulatory effects; however, its single administration is hindered by inadequate BBB penetration and weak single-agent anti-glioma potency (7). Cabazitaxel (CTX), a second-generation taxane chemotherapeutic agent, effectively inhibits tumor proliferation and migration by disrupting microtubule dynamics; however, free CTX is limited by rapid systemic clearance, severe off-target toxicity, and inadequate intracranial enrichment following intravenous administration (8). The combination therapy of ELE and CTX can merge the mild immune-modulating characteristics of herbal monomers with the potent cytotoxicity of chemotherapeutics to produce synergistic antitumor effects. However, free co-administration still fails to overcome the dual obstacles of BBB impermeability and tumor non-specific distribution. To address this limitation, we previously developed glioma cytomembrane-coated liposomes co-loaded with ELE and CTX to improve intracranial drug administration; however, these tumor cell-derived membranes are susceptible to macrophage elimination and induce unwanted immune responses, thereby limiting their *in vivo* therapeutic efficacy (9). Therefore, we optimized the biomimetic coating strategy with genetically engineered cell membranes to further advance glioma-targeted chemoimmunotherapy.

Recent studies have demonstrated that mesenchymal stem cells (MSCs) naturally migrate to gliomas and readily cross the BBB, offering superior specificity for brain tumor targeting (10). Similar to embryonic stem cells, MSCs can undergo epigenetic programming to express desired surface proteins, thereby acquiring immune-regulatory functions tailored to immunotherapy (11). Autologous MSCs minimize immunogenicity, hence facilitating clinical translation. Cloaking nanoparticles with engineered MSC membranes simultaneously confers BBB-crossing capability, glioma tropism, and immunomodulatory activity, overcoming the intrinsic limitations of conventional nanocarriers and facilitating a definitive translational approach for glioma treatment (10, 12).

Although MSC-membrane-coated nanoparticles readily penetrate the BBB and migrate to glioma lesions, they encounter challenges of inadequate specific retention within tumor tissues post-intracranial delivery. This unmet tumor enrichment limitation constitutes a lingering therapeutic obstacle (13, 14). To address inadequate intratumoral accumulation and simultaneously reverse tumor immunosuppression, we genetically engineered MSCs to overexpress programmed cell death protein 1 (PD-1) on their membrane surface. The MSC-derived shell confers BBB penetration and brain tropism, whereas surface-displayed PD-1 provides two additional unique functions: first, PD-1 specifically binds overexpressed PD-L1 on glioma cells through ligand-receptor interaction to boost active tumor targeting and intratumoral nanoparticle retention; second, the free PD-1 on the nanoparticle surface competitively inhibits the PD-1/PD-L1 immune checkpoint pathway, alleviating CD8+ T cell exhaustion and remodeling the immunosuppressive TME (15).

Here, we proposed the following hypothesis: developing PD-1-functionalized MSC membrane biomimetic nanoparticles (PD-1-MSCs-ELE/CTX@BLIP) promotes efficient drug penetration through the BBB to achieve antitumor effects; it specifically targets PD-L1+ glioma cells through the PD-1/PD-L1 axis, competitively occupies PD-L1 binding sites, prevents CD8+ T cell exhaustion, and consequently modifies the antitumor immune response (Graphical Abstract). First, we delineated PD-1-functionalized MSCs and extracted their cell membranes. Subsequently, we improved the previously established method for preparing the ELE/CTX lipidosome and combined it with the ultrasonic treatment method to prepare PD-1-MSCs-ELE/CTX@BLIP. BBB permeability, *in vitro* and *in vivo* targeting, and pharmacodynamic studies were conducted using glioma models to further clarify the anti-glioma effect of PD-1-MSCs-ELE/CTX@BLIP. Overall, this study clarified the mechanism by which PD-1-MSCs-ELE/CTX@BLIP crosses the BBB and reshapes CD8+ T cell-mediated anti-glioma immunity.

## 2 Materials and methods

### 2.1 Materials

Elemene (ELE) was purchased from Dalian Huali Jingang Pharmaceutical Co., Ltd. (Dalian, China). Cabazitaxel (CTX) was obtained from Hubei Qianmo Biological Technology Co., Ltd. (Hubei, China). Phosphatidylcholine (Pc) and cholesterol (Chol) were purchased from Shanghai Lieneng Technology Development Co., Ltd. (Shanghai, China) and Chengdu Kelong Chemicals Co., Ltd. (Chengdu, China), respectively. Chloroform was supplied by Sichuan Xiling Science Co., Ltd. (Chengdu, China). Penicillin–streptomycin, high-glucose DMEM, trypsin-EDTA, and fetal bovine serum (FBS) were obtained from Gibco Life Technologies (AG, USA). The Cell Counting Kit-8 (CCK-8) was purchased from Meilunbio (Dalian, China). Coomassie blue and the bicinchoninic acid (BCA) protein assay kit were obtained from Biosharp (Shanghai, China). Dimethyl sulfoxide (DMSO) was purchased from Sigma-Aldrich Trading Co., Ltd. (Shanghai, China). Isoflurane was sourced from Shandong Ante Livestock Technology Co., Ltd. (Jinan, China). Phosphate-buffered saline (PBS), RIPA lysis buffer, 4% paraformaldehyde tissue fixative, and optimal cutting temperature (OCT) embedding compound (for frozen sections) were purchased from Wuhan Servicebio Biotechnology Co., Ltd. (Wuhan, China). Anti-fade mounting medium containing DAPI, a cell cycle detection kit, and an apoptosis detection kit were obtained from Shanghai Beyotime Biotechnology Co., Ltd. (Shanghai, China).

### 2.2 Cell culture

G422 glioma cells (Cell Resource Center, Institute of Basic Medical Sciences, CAMS/PUMC, Beijing, China) and bEnd.3 cells (Chinese Academy of Sciences, Shanghai, China) were cultured in high-glucose (4.5 g/L) DMEM supplemented with 10% FBS, 1% penicillin-streptomycin, 110 mg/l sodium pyruvate, and 2 mM l-glutamine and were incubated in normoxic conditions (5% CO_2_ and 37 °C). Mesenchymal stem cells (MSCs, Wuhan Pricella Biotechnology Co., Ltd., Wuhan, China) were cultured in αMEM supplemented with 10% FBS, 1% penicillin-streptomycin and were incubated in normoxic conditions (5% CO_2_ and 37 °C) (16).

### 2.3 Construction and isolation of PD-1-MSCs

To generate PD-1-overexpressing MSCs (PD-1-MSCs), PD-1+ MSCs were established using lentiviral infection followed by puromycin selection (**Supplementary Table 1**, Supporting Information **Supplementary Figure 1**). Briefly, the vector and PD-1 target gene fragments underwent double digestion, separated and recovered using electrophoresis, and subsequently ligated overnight at room temperature using T4 DNA ligase. The mixture was converted into DH5α competent cells and cultured on antibiotic agar plates at 37 °C for 12 h. Single colonies were selected and amplified to extract recombinant plasmids. Lentivirus was encapsulated within 293T cells and subsequently concentrated. Furthermore, 2 × 10^4^ cells were seeded in 96-well plates. When cell confluence reached 60%–70%, a serial dilution of the virus mixed with 8 µg/mL polybrene was added. Cells were cultured for 24 h before medium renewal, incubated for another 48 h, and green fluorescent protein (GFP)-positive cells were counted under a microscope to calculate viral titer. Additionally, 2 × 10^3^ MSCs were seeded in 24-well plates and cultured for 12 h to reach ~20% confluence, followed by 12 h co-incubation with PD-1 lentivirus and polybrene. Obvious green fluorescence was observed at 72 h post-transfection, followed by the addition of 2 μg/mL puromycin for continuous screening to eliminate negative cells until all cells expressed GFP, and the PD-1 stably overexpressed MSC line was obtained (15). PD-1 expression and membrane localization were confirmed by co-localization with GFP-tagged PD-1. PD-1 expression was further confirmed using quantitative polymerase chain reaction (qPCR), flow cytometry (FACS), Western blotting (WB), and confocal laser scanning microscopy (CLSM; TCS SP8 STED, Leica, Germany) (15).

PD-1-MSC membranes (PD-1-MSCsM) were extracted as follows. Cells were harvested and resuspended in 5 mL of cell lysis buffer (containing 0.25 M sucrose, 1 mM EDTA, 20 mM Hepes-NaOH, and protease inhibitors, pH 7.4). The suspension was placed on ice and subjected to 8 cycles of ultrasonication using a Selecta Sonopuls cell disruptor (3 s on, 7 s off per cycle). The mixture was centrifuged at 3000 × g for 5 min, and the supernatant was collected. The supernatant was subsequently centrifuged at 14,000 × g for 20 min at 4 °C to isolate pure PD-1-MSCsM, which were subsequently resuspended in 1× phosphate-buffered saline (PBS) and extruded through 0.45 µm pores. Protein concentration was assessed utilizing a bicinchoninic acid protein assay for subsequent liposome preparation. PD-1-MSCsM obtained from 1 × 10^8^ PD-1-MSCs weighed 0.50 mg and were stored at −80 °C (17, 18).

### 2.4 Preparation and characterization of PD-1-MSCs-ELE/CTX@BLIP

Pc (84 mg) and Chol (16 mg) were solubilized in 20 mL of chloroform with thorough stirring. Subsequently, CTX (2 mg) and ELE (20 mg) were added, and the mixture was ultrasonicated for 15 min until complete dissolution. The organic solvent was removed using a rotary evaporator. Following the establishment of a homogeneous film on the inner wall of the round-bottom flask, evaporation persisted for 2 h to completely remove residual solvent. The film was hydrated with 10 mL of PBS (pH 7.4) for 1 h, followed by probe ultrasonication for 10 min (5 s on, 5 s off). The resulting dispersion was filtered through a 0.45 µm filter membrane to obtain liposomes encapsulating ELE/CTX (ELE/CTX@LIP). Subsequently, ELE/CTX@LIP was mixed with PD-1-MSCsM (the mass ratio range of PD-1-MSCsM and phospholipids is 0.5-4:1), and probe ultrasonication (150 W, 5 min, 3 s on, 3 s off) was utilized to enhance encapsulation/fusion, yielding PD-1-MSCs-ELE/CTX@BLIP (19). The encapsulation efficiency (EE) and drug loading efficiency (DLE) of ELE and CTX were assessed using HPLC analysis (LC-45202-46, SHIMADZU, JAPAN). EE was subsequently determined by the equation: EE = (Wa/Wa + Wb) × 100% and DLE = [Wa/Wt] × 100%, where Wa represents the amount of ELE or CTX encapsulated in liposomes after hyperfiltration, Wb represents the amount of ELE or CTX unencapsulated, and Wt represents the total amount of phospholipid (9).

The morphology of PD-1-MSCs-ELE/CTX@BLIP was examined using transmission electron microscopy (TEM; JEM-1200X, JEOL, Japan). The size distribution and ζ-potential were assessed by dynamic light scattering (DLS; Litesizer 500, Anton Paar, Austria). Storage stability in PBS was evaluated by monitoring particle size changes over 15 days using DLS. Protein profiles of PD-1-MSCsM and PD-1-MSCs-ELE/CTX@BLIP were analyzed using sodium dodecyl sulfate–polyacrylamide gel electrophoresis (SDS-PAGE). Liposomes were stained with DiD and fused with GFP-tagged PD-1-MSCsM. The core–shell structure was observed using CLSM. Encapsulation efficiency (EE) and drug loading efficiency (DLE) of ELE and CTX were determined using ultra-performance liquid chromatography (UPLC; LC-45202-46, Shimadzu, Japan) as described previously (9). Overall, the safety of PD-1-MSCs-ELE/CTX@BLIP was evaluated using a hemolysis test.

### 2.5 *In vitro* study of BBB permeability and glioma cell targeting uptake

A Transwell model was employed to evaluate the ability of PD-1-MSCs-ELE/CTX@BLIP to penetrate the BBB and its therapeutic effect on G422 cells. Additionally, bEnd.3 cells (1 × 10^5^) were seeded in the upper chamber of the Transwell insert, whereas the lower chamber was filled with Dulbecco’s modified Eagle medium supplemented with 10% fetal bovine serum. Experiments were performed after the transendothelial electrical resistance value had stabilized. Free drugs, ELE/CTX@LIP, MSCs-ELE/CTX@BLIP, and PD-1-MSCs-ELE/CTX@BLIP were added to the upper chamber. After 4 h of incubation, the ELE/CTX concentration in the lower chamber was measured utilizing an SP8 STED ultra-high-resolution imaging system (Leica, Germany) and UPLC to evaluate BBB penetration. Separately, cell slides were placed in the lower chamber and seeded with 1 × 10^5^ G422 cells. Free DiD, DiD@LIP, MSC-DiD@BLIP, and PD-1-MSC-DiD@BLIP (DiD concentration: 50 ng/mL) were introduced into the upper chamber. After 4 h, the Transwell insert was removed, and the cells were stained with DAPI for 15 min. The cellular absorption by G422 cells was visualized using CLSM and quantitatively analyzed using FACS (20, 21).

For direct uptake investigations, PD-1-MSCs-ELE/CTX@BLIP was incubated with G422 cells or 4T1 cells. Cells were seeded in 6-well plates (3 × 10^5^ cells/well) and cultured for 24 h. The medium was subsequently replaced with fresh medium containing PD-1-MSCs-ELE/CTX@BLIP (DiD concentration: 50 ng/mL) and incubated for 2 h. In separate experiments, G422 cells were seeded in 6-well plates (3 × 10^5^ cells/well) and incubated with free DiD (DiD concentration: 50 ng/mL), ELE/CTX@LIP, MSCs-ELE/CTX@BLIP, PD-1-MSCs-ELE/CTX@BLIP, or PD-1-MSCs-ELE/CTX@BLIP plus a PD-L1 inhibitor (C_25_H_29_N_3_O_3_, Macklin) for 2 h. The medium was removed, and the cells were fixed with 4% paraformaldehyde, stained with DAPI, and subsequently washed three times with PBS. Cellular uptake was determined using CLSM and FACS (9).

### 2.6 *In vitro* cytotoxicity assay and cell apoptosis

The cytotoxicity of free drugs, ELE/CTX@LIP, MSCs-ELE/CTX@BLIP, and PD-1-MSCs-ELE/CTX@BLIP against G422 cells was evaluated using a Cell Counting Kit-8 kit. G422 cells (3,000 cells/well) were seeded in 96-well plates and incubated for 24 h. The cells were subsequently exposed to the indicated formulations (CTX concentrations ranging from 10 to 80 ng/mL) for 48 h. Untreated cells served as negative controls, and culture medium was utilized as the blank control. Cytotoxicity was quantified by measuring absorbance at 450 nm using a Spark multimode microplate reader (Tecan, Switzerland). Cellular migration and repair capabilities were assessed using the scratch wound healing assay (22).

Apoptosis was analyzed using flow cytometry (FACSVerse, BD Biosciences, USA). G422 cells were incubated with free drugs, ELE/CTX@LIP, MSCs-ELE/CTX@BLIP, or PD-1-MSCs-ELE/CTX@BLIP (CTX 20 ng/mL) for 48 h and subsequently treated with an apoptosis detection kit for 10 min. The percentage of apoptotic cells was determined utilizing a FACS Calibur system. Untreated cells served as negative controls. The levels of interleukin (IL)-6, tumor necrosis factor (TNF)-α, and IL-1β were quantified using enzyme-linked immunosorbent assay (ELISA) to characterize the therapeutic effect of PD-1-MSCs-ELE/CTX@BLIP on glioma (23).

### 2.7 Animal and glioma-bearing model

Female BALB/c mice (20 ± 2 g) were procured from Shanghai Slack Laboratory Animal Co., Ltd. The mice were acclimatized at room temperature for 7 days before the start of the experiments. Animals were maintained in an animal care facility with ad libitum access to food and water. All animal experiments were approved by the Animal Ethics Committee of Chengdu University of Traditional Chinese Medicine (Chengdu, China). For the subcutaneous glioma model, G422 cells were mixed with PBS to prepare a cell suspension (1 × 10^7^ cells/mL). Under sterile conditions, 100 μL of the suspension was injected subcutaneously into the right flank of 4–6-week-old wild-type BALB/c mice (24, 25). Tumor proliferation was monitored regularly after inoculation, and tumor volume was measured every 2 days using the formula V = length × width^2^/ 2. When the tumor volume reached 50–100 mm^3^, the mice were randomly assigned to treatment groups (26, 27). Each experimental group was assigned 8 mice. All included mice were ensured to possess approximately the same tumor size at the initial stage. Tumors above 100 mm³ and below 50 mm^3^ were excluded.

For the orthotopic glioma model, female BALB/c mice (20 ± 2 g) were procured from Sibefu (Beijing) Biotechnology Co., Ltd. The mice were anesthetized through intraperitoneal injection of 10% chloral hydrate, and their heads were immobilized in a stereotaxic frame. Approximately 2.5 × 10^7^ G422 cells suspended in medium were slowly injected into the right striatum (coordinates: 2.0 mm lateral, 1.8 mm anterior to bregma, 3.5 mm depth). The needle was kept in place for 1 min after injection and was slowly withdrawn subsequently. The skin was disinfected with an alcohol swab and sutured. The mice were returned to their cages to recover naturally. Intracranial tumor growth was monitored by fluorescence imaging (28, 29). For the *in situ* model, the tumor size was evaluated using fluorescence quantification. The inclusion and exclusion criteria were similar to those of the subcutaneous tumor model.

### 2.8 *In vivo* fluorescence imaging assay

A subcutaneous glioma-bearing model was established by injecting G422-Luc cells into the right flank as described above. Free DiD, DiD@LIP, MSCs-DiD@BLIP, and PD-1-MSCs-DiD@BLIP (DiD dose: 0.1 mg/kg) were injected into glioma-bearing mice through the tail vein. Fluorescence imaging was performed at predetermined time points (3, 6, 12, 24, and 48 h) using an *in vivo* imaging system (IVIS Spectrum, PerkinElmer). These mice were subsequently euthanized, and the tumors, together with the heart, liver, spleen, lung, and kidney, were harvested for quantitative biodistribution analysis and *ex vivo* fluorescence imaging (30).

### 2.9 *In vivo* antitumor study

Mice with orthotopic glioma were randomly divided into six groups (n = 6 per group) (1): control (physiological saline) (2); free drugs (ELE + CTX) (3); ELE/CTX@LIP (4); MSCs-ELE/CTX@BLIP (5); PD-1-MSCs-ELE/CTX@BLIP (6); PD-L1 inhibitor. Treatments were administered through intravenous injection into the tail vein on days 1, 3, 5, 7, 9, and 11. Groups 2–6 received a first dose of CTX at 2.5 mg/kg, followed by five subsequent injections of 0.625 mg/kg CTX. Fluorescence imaging was performed on days 1, 5, 10, and 15 to observe tumor proliferation. Body weight and survival duration were recorded every 3 days. Mice were euthanized 2 h after the final treatment, and their brains were collected for TUNEL immunofluorescence staining (28, 29).

The subcutaneous glioma-bearing model was established as previously outlined. Grouping and treatment regimens were the same as those for the orthotopic model. Tumor tissues were excised, weighed, and homogenized. Levels of TNF-α, interferon (IFN)-γ, and IL-1β in the TME were quantified using ELISA to evaluate the therapeutic effect of PD-1-MSCs-ELE/CTX@BLIP on glioma (26, 27).

### 2.10 Toxicity evaluation

Glioma-bearing mice were divided into six groups and treated with (1) control (physiological saline) (2), free drugs (3), ELE/CTX@LIP (4), MSCs-ELE/CTX@BLIP (5), PD-1-MSCs-ELE/CTX@BLIP, or (6) PD-L1 inhibitor. Treatments were administered every other day for six doses. Whole blood samples were collected from the retro-orbital sinus 2 h after the final treatment. A portion of each sample was utilized for complete blood count analysis. The remaining blood was centrifuged (4,000 × g, 10 min) to obtain serum, which was utilized to measure biochemical indices of liver and kidney function, including total bilirubin, blood urea nitrogen, uric acid, alanine aminotransferase (ALT), creatinine, and aspartate aminotransferase (AST), to evaluate the toxicity of free drugs and liposome formulations (31).

### 2.11 Statistical analysis

Statistical analysis was performed using GraphPad Prism software (version 10.4.0; GraphPad Software Inc., San Diego, CA, USA). Data are presented as mean ± standard deviation. Comparisons between two groups were analyzed using a two-tailed Student’s t-test, and comparisons among multiple groups were performed using one-way or two-way analysis of variance as appropriate. Statistical significance was defined as *P < 0.05, and highly significant differences were indicated by **P < 0.01, ***P < 0.001, or ****P < 0.0001.

## 3 Results

### 3.1 Construction and characterization of PD-1-MSCs

**Figure 1:**
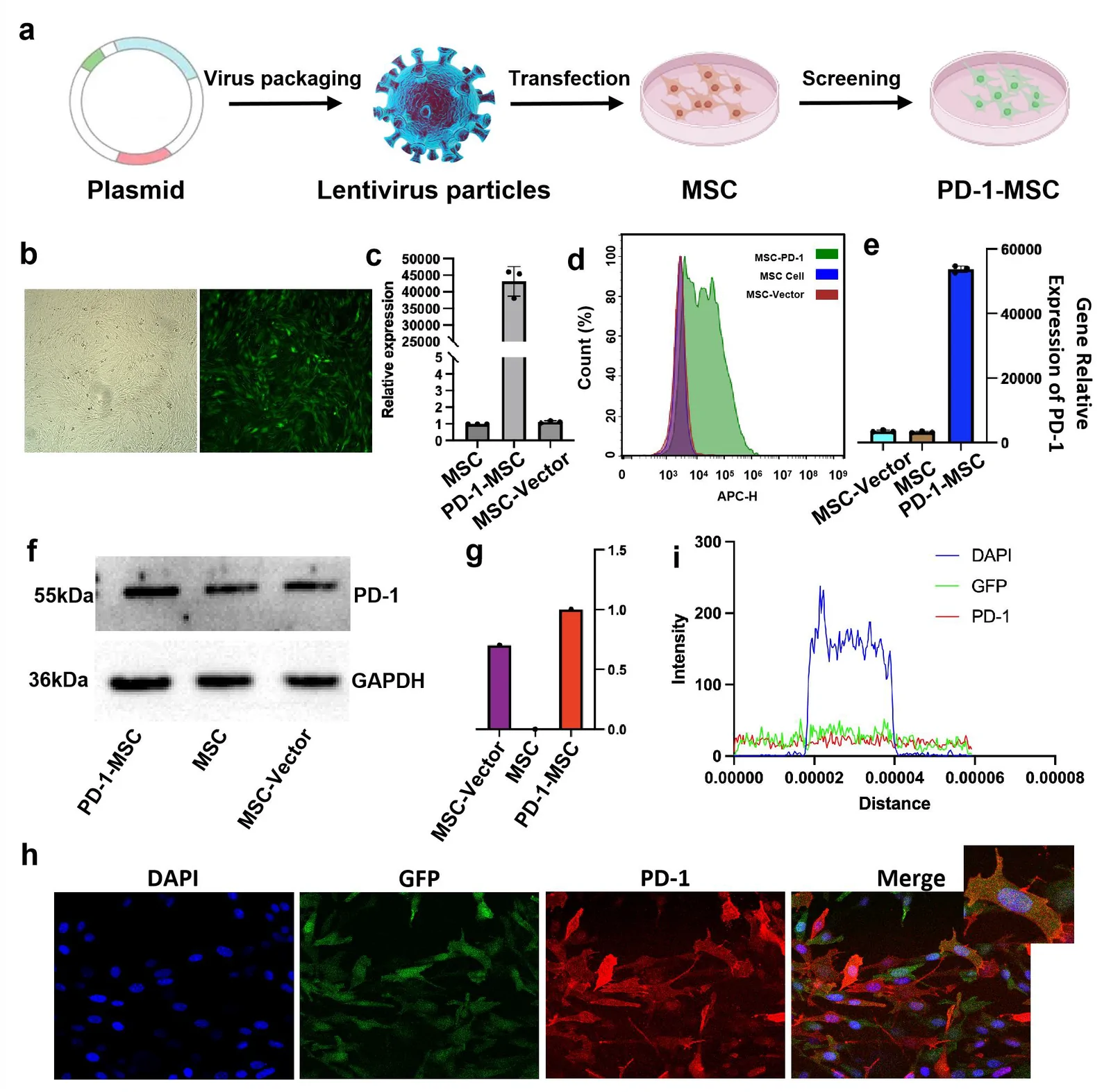
Construction and Characterization of PD-1-MSCs. **(A)** Schematic illustration of PD-1-MSCs generation. **(B)** GFP expression in PD-1-MSCs observed under fluorescence microscopy. **(C)** PD-1 mRNA quantification by Q-PCR (n = 3). **(D)** Flow-cytometric analysis of PD-1 surface expression. **(E)** Fluorescence-based quantitative assessment of PD-1 expression (n = 3). **(F)** WB detection of PD-1 protein. **(G)** Quantitative analysis of PD-1 protein levels (n = 3). **(H)** Immunofluorescence visualization of PD-1 membrane localization. **(I)** Fluorescence-intensity profile along the membrane.

To generate PD-1-overexpressing MSCs (PD-1-MSCs), we established PD-1+ MSCs through lentiviral infection followed by puromycin selection (**Figure 1A**) (32). PD-1 expression and its localization to the plasma membrane were confirmed by co-localization with GFP-tagged PD-1 (**Figure 1B**, Supporting Information **Supplementary Figure 2**), demonstrating successful lentiviral transduction of MSCs and efficient PD-1 gene expression in the engineered cells (15). Non-transfected MSCs revealed no green fluorescence (Supporting Information **Supplementary Figure 1** and **2**). PD-1 expression was further validated using qPCR, flow cytometry (FACS), WB, and CLSM (**Figures 1C–H**). Compared with untransfected MSCs, PD-1-MSCs demonstrated a > 40,000-fold increase in PD-1 mRNA (**Figure 1C**) and a > 50,000-fold increase in PD-1 protein levels (**Figures 1D, E**). These observations indicate that PD-1 expression was significantly increased in PD-1-MSCs at the transcriptional and translational levels, further confirming the effective transfer of the PD-1 gene into MSCs (33). WB results confirmed these findings (**Figures 1F, G**) (15). Subsequent investigations confirmed that PD-1 was predominantly localized to the MSC membrane surface (**Figures 1H, I**).

### 3.2 Preparation and characterization of PD-1-MSCs-ELE/CTX@BLIP

**Figure 2:**
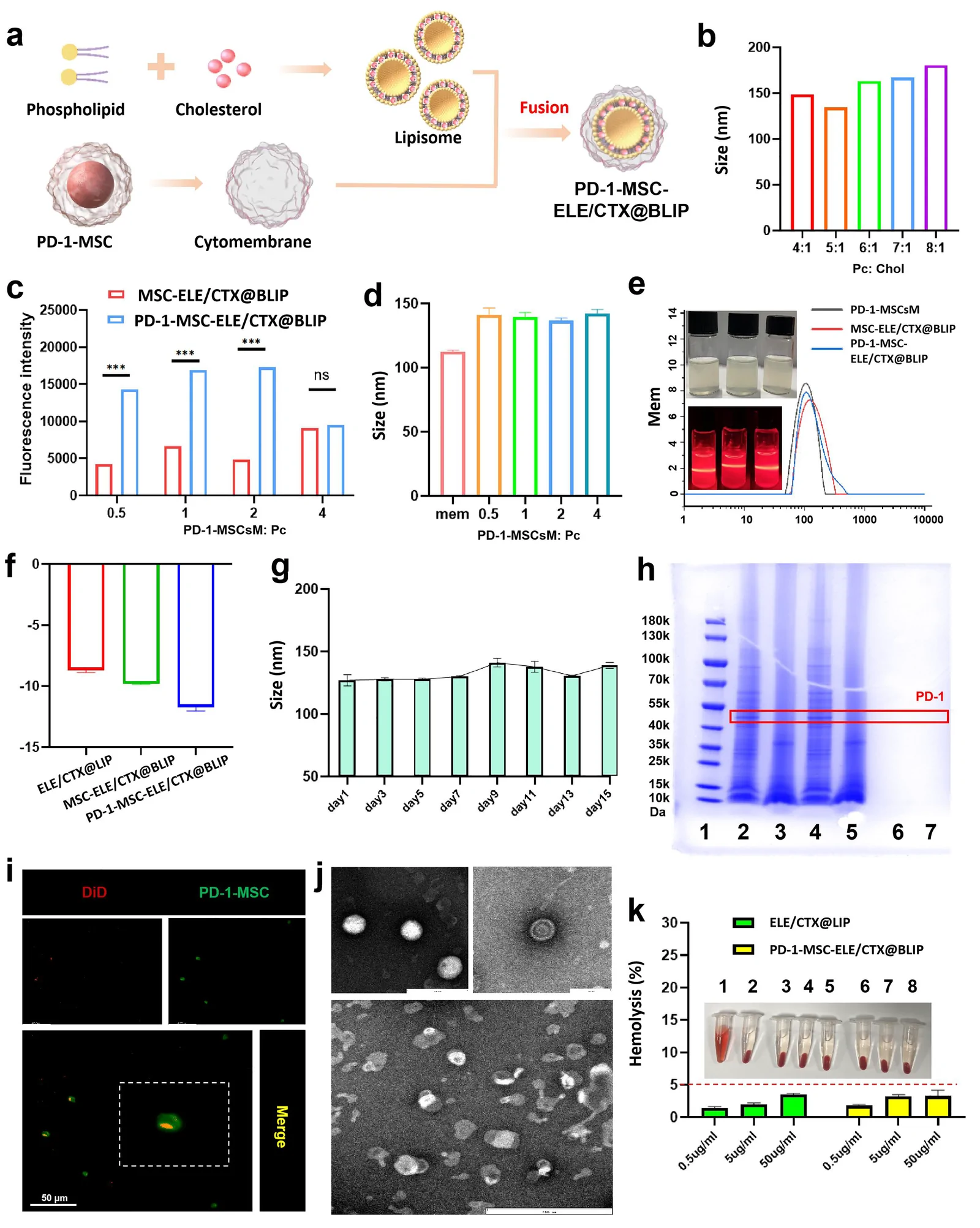
Construction and property evaluation of PD-1-MSCs-ELE/CTX@BLIP. **(A)** Schematic illustration of the assembly of PD-1-MSCs-ELE/CTX@BLIP. **(B)** Optimization of the phospholipid-to-cholesterol ratio. **(C)** Optimization of the phospholipid-to-membrane ratio—the fluorescence uptake amount of G422 cells determined by flow cytometry. **(D)** Optimization of the phospholipid-to-membrane ratio—particle size. **(E)** Representative photograph and size distribution of the formulation. **(F)** ζ-Potential (n = 3). **(G)** Particle-size stability over 15 days (n = 3). **(H)** SDS-PAGE results (1): Marker (2), PD-1-MSCsM (3), MSCs (4), PD-1-MSCs-ELE/CTX@BLIP (5), MSCs-ELE/CTX@BLIP (6), ELE/CTX@LIP (7), Free. **(I)** Confocal verification of membrane–nanoparticle fusion. Scale, 50 μm. **(J)** TEM image. **(K)** *In vitro* hemocompatibility (1): Water (2), Normal saline (3), ELE/CTX@LIP at 0.1 μg/mL (4), ELE/CTX@LIP at 1 μg/mL (5), ELE/CTX@LIP at 10 μg/mL (6), PD-1-MSCs-ELE/CTX@BLIP at 0.1 μg/mL (7), PD-1-MSCs-ELE/CTX@BLIP at 1 μg/mL (8), PD-1-MSCs-ELE/CTX@BLIP at 10 μg/mL (n = 3). ***P < 0.001.

PD-1-MSCs-ELE/CTX@BLIP was obtained by enveloping pre-formed ELE/CTX@LIP with PD-1-MSC membranes (PD-1-MSCsM) (**Figure 2A**) (9). Before final assembly, we refined the ELE/CTX@LIP preparation protocol, including the phospholipid-to-cholesterol ratio and the quantity of PD-1-MSCsM. A phospholipid-to-cholesterol ratio of 5:1 yielded ELE/CTX@LIP with the most favorable particle size (**Figure 2B**). Increasing the quantity of PD-1-MSCsM gradually enhanced the uptake of PD-1-MSCs-ELE/CTX@BLIP by G422 cells compared with ELE/CTX@LIP, although no significant difference was observed within the tested concentration range. The optimal ratio is illustrated in **Figures 2C, D**. The comprehensive preparation procedure for PD-1-MSCs-ELE/CTX@BLIP is provided in Section 2.4. Visual inspection revealed that PD-1-MSCs-ELE/CTX@BLIP samples were opalescent, translucent liquids (**Figure 2E**). The mean particle size, polydispersity index (PDI), and ζ-potential measured 135.85 nm, 0.182, and −11.72 mV, respectively (**Figure 2F**; **Supplementary Table 2**), confirming the uniformity and colloidal stability of the formulation. **Supplementary Table 2** presents corresponding parameters for ELE/CTX@LIP and MSCs-ELE/CTX@BLIP. The encapsulation efficiencies of ELE and CTX in PD-1-MSCs-ELE/CTX@BLIP attained 98.5% and 94.2%, respectively, with drug loading capacities of 15.9% and 1.5% (**Supplementary Table 2**). Following a 15-day storage period at 4 °C, no significant change in particle size or ζ-potential was observed, indicating excellent storage stability and low hygroscopicity (**Figure 2G**, Supporting Information **Supplementary Figure 3**) (34). These data demonstrate that PD-1-MSCs-ELE/CTX@BLIP is suitable for subsequent *in vitro* and *in vivo* studies. SDS-PAGE was performed to compare the protein profiles of PD-1-MSCs-ELE/CTX@BLIP, MSCs-ELE/CTX@BLIP, ELE/CTX@LIP, PD-1-MSCs, MSCs, and free drugs. The protein pattern of PD-1-MSCs-ELE/CTX@BLIP matched that of PD-1-MSCsM, and the distinct PD-1 band distinguished it from MSCs-ELE/CTX@BLIP and MSCs (**Figure 2H**). Confocal microscopy revealed that the liposomes were securely enclosed by PD-1-MSCsM, validating the successful assembly (**Figure 2I**). TEM images (**Figure 2J**, Supporting Information **Supplementary Figure 4**) demonstrated that the PD-1-MSCsM contained within them resulted in the formation of spherical nanostructures with irregular surfaces and a clear core-shell structure. Furthermore, PD-1-MSCs-ELE/CTX@BLIP induced negligible hemolysis (< 5%), highlighting its favorable biosafety profile (**Figure 2K**) (35).

### 3.3 *In vitro* targeting of PD-1-MSCs-ELE/CTX@BLIP

**Figure 3:**
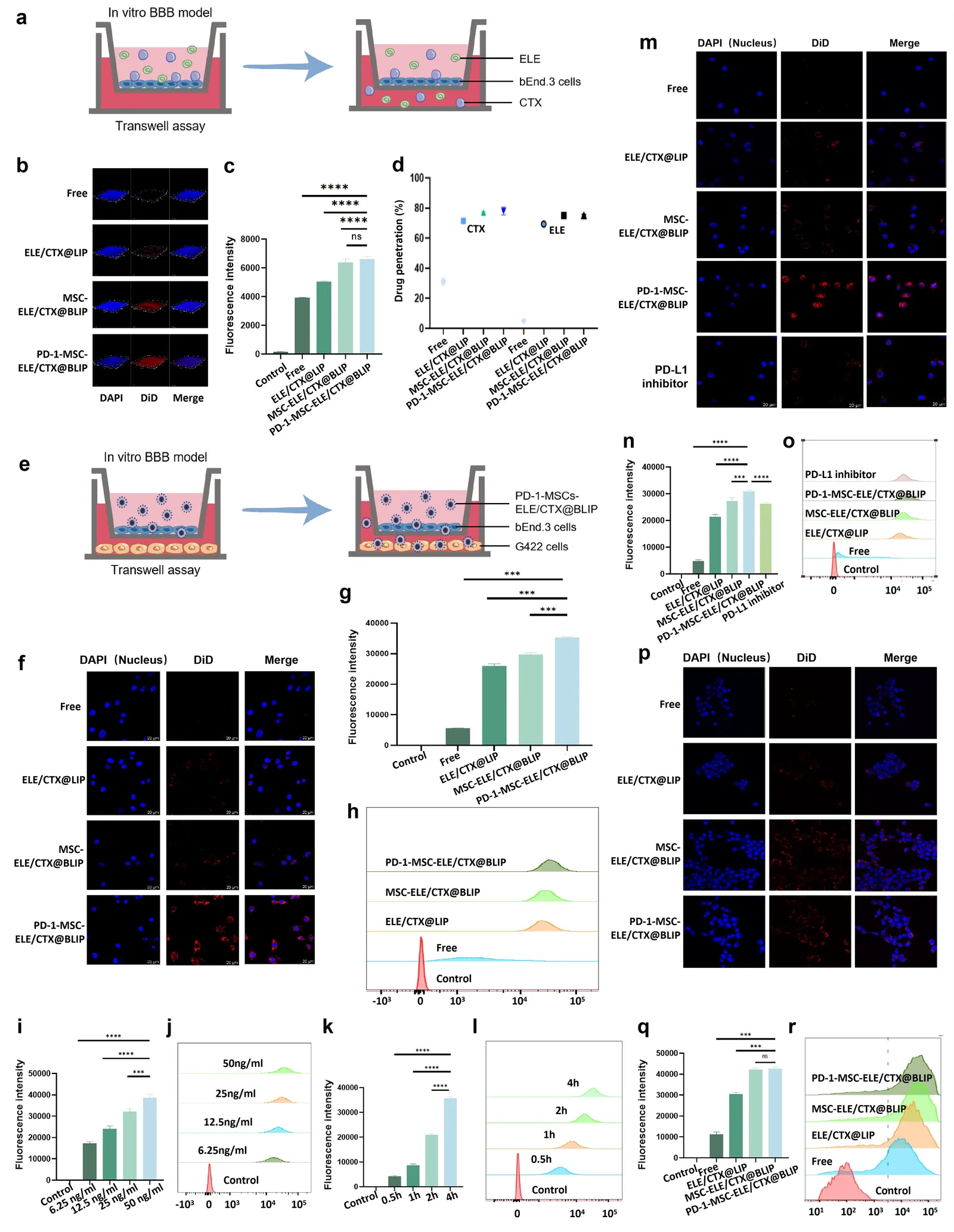
Evaluation of *in vitro* BBB-crossing capacity and glioma-cell targeting. **(A)** Schematic illustration of the *in vitro* BBB model. **(B)** HIS-SIM was used to characterize the intensity of DiD fluorescence uptake in bEnd.3 cells. **(C)** Quantitative determination of DiD fluorescence uptake intensity in bEnd.3 cells (n = 3). **(D)** UPLC determination of the drug content that had passed through the BBB (n = 3). **(E)** Schematic illustration of the *in vitro* BBB-crossing and targeting-uptake evaluation. **(F)** CLSM images and fluorescence quantification of G422 cells treated with different formulations for 2 h in the *in vitro* BBB transwell assay. **(G)** Fluorescence quantification of G422 cells treated with different formulations for 2 h in the *in vitro* BBB transwell assay (n = 3). **(H)** Fluorescence quantitative analysis corresponding to FACS in the *in vitro* BBB transwell assay. **(I)** Fluorescence quantification of G422 cells treated with different concentrations of PD-1-MSCs-ELE/CTX@BLIP (n = 3). **(J)** Fluorescence quantitative analysis corresponding to FACS at different concentrations of PD-1-MSCs-ELE/CTX@BLIP. **(K)** Fluorescence quantification of G422 cells treated with PD-1-MSCs-ELE/CTX@BLIP for different incubation times (n = 3). **(L)** Fluorescence quantitative analysis corresponding to FACS at different incubation times of PD-1-MSCs-ELE/CTX@BLIP. **(M)** CLSM images and fluorescence quantification of PD-L1+ glioma cells treated with PD-1-MSCs-ELE/CTX@BLIP. **(N)** Fluorescence quantification of PD-L1+ glioma cells treated with PD-1-MSCs-ELE/CTX@BLIP (n = 3). **(O)** Fluorescence quantitative analysis corresponding to FACS of PD-1-MSCs-ELE/CTX@BLIP in PD-L1+ glioma cells. **(P)** CLSM images and fluorescence quantification of PD-L1-glioma cells treated with PD-1-MSCs-ELE/CTX@BLIP. **(Q)** Fluorescence quantification of PD-L1-glioma cells treated with PD-1-MSCs-ELE/CTX@BLIP (n = 3). **(R)** Fluorescence quantitative analysis corresponding to FACS of PD-1-MSCs-ELE/CTX@BLIP in PD-L1- glioma cells. ***P < 0.001; ****P < 0.0001; ns, not significant.

An *in vitro* BBB model was established utilizing bEnd.3 cells to evaluate the permeability and targeting capacity of PD-1-MSCs-ELE/CTX@BLIP (**Figure 3A**) (36). High-sensitivity structured illumination microscope (HIS-SIM) revealed that the DiD fluorescence signal in the PD-1-MSCs-ELE/CTX@BLIP group was significantly stronger than that in the other groups, indicating substantially improved drug penetration across the BBB (**Figure 3B**). FACS analysis further corroborated these findings (**Figure 3C**). The amount of ELE/CTX that crossed the BBB was subsequently quantified using UPLC. The findings revealed that encapsulation of drugs in liposomes enhanced conventional BBB permeability (**Figure 3D**) (37, 38), and this permeation was further augmented by cell membrane cloaking. FACS and CLSM were subsequently employed to evaluate the specific absorption of PD-1-MSCs-ELE/CTX@BLIP by G422 cells following BBB transit (**Figure 3E**). Qualitative imaging revealed that the fluorescence intensity in the PD-1-MSCs-ELE/CTX@BLIP group was significantly higher than in the other groups, suggesting increased drug accumulation in glioma cells (**Figure 3F**). Quantitative FACS analysis revealed that DiD absorption by G422 cells in the PD-1-MSCs-ELE/CTX@BLIP group was 6.26-, 1.35-, and 1.17-fold higher than that in the free drug, ELE/CTX@LIP, and MSCs-ELE/CTX@BLIP groups, respectively, hence confirming enhanced drug accumulation in glioma cells (**Figures 3G, H**). These data demonstrate that PD-1-MSCs-ELE/CTX@BLIP promotes drug absorption by glioma cells after BBB crossing, an effect further amplified by PD-1 overexpression (39, 40).

We further evaluated the specific uptake capacity of PD-1-MSCs-ELE/CTX@BLIP to verify its safety and selectivity. First, we compared absorption at different DiD concentrations and incubation times. FACS analysis revealed that fluorescence intensity in G422 cells increased significantly with increasing DiD concentration and incubation time (**Figures 3I–L**). Accordingly, a DiD concentration of 50 ng/mL and an incubation time of 4 h were selected for subsequent targeting studies (41). Under these conditions, drug delivery efficiency to G422 cells was evaluated using CLSM and FACS across different formulations. The red fluorescence intensity in the PD-1-MSCs-ELE/CTX@BLIP group was significantly higher than that in the free drug, ELE/CTX@LIP, and MSCs-ELE/CTX@BLIP groups, signifying superior delivery efficiency (**Figure 3M**). Upon administration of a PD-L1 inhibitor, this advantage was abolished, and delivery efficiency became comparable to that of the MSCs-ELE/CTX@BLIP group, confirming that the enhanced absorption of G422 cells is mediated by the PD-1/PD-L1 axis (**Figures 3N, O**) (42, 43). This PD-1/PD-L1-dependent targeting selectively delivers therapeutics to PD-L1+ glioma cells and spares normal tissues. Similarly, we examined delivery to 4T1 cells (PD-L1–). The results revealed that PD-1-MSCs-ELE/CTX@BLIP uptake did not differ significantly from that of MSCs-ELE/CTX@BLIP but remained significantly higher than that of the free drug and ELE/CTX@LIP groups, reaffirming the superiority of MSC-biomimetic nanoparticles and the indispensable role of the PD-1/PD-L1 axis in enhancing delivery efficiency (**Figures 3P–R**).

### 3.4 *In vitro* antitumor efficacy of PD-1-MSCs-ELE/CTX@BLIP

**Figure 4:**
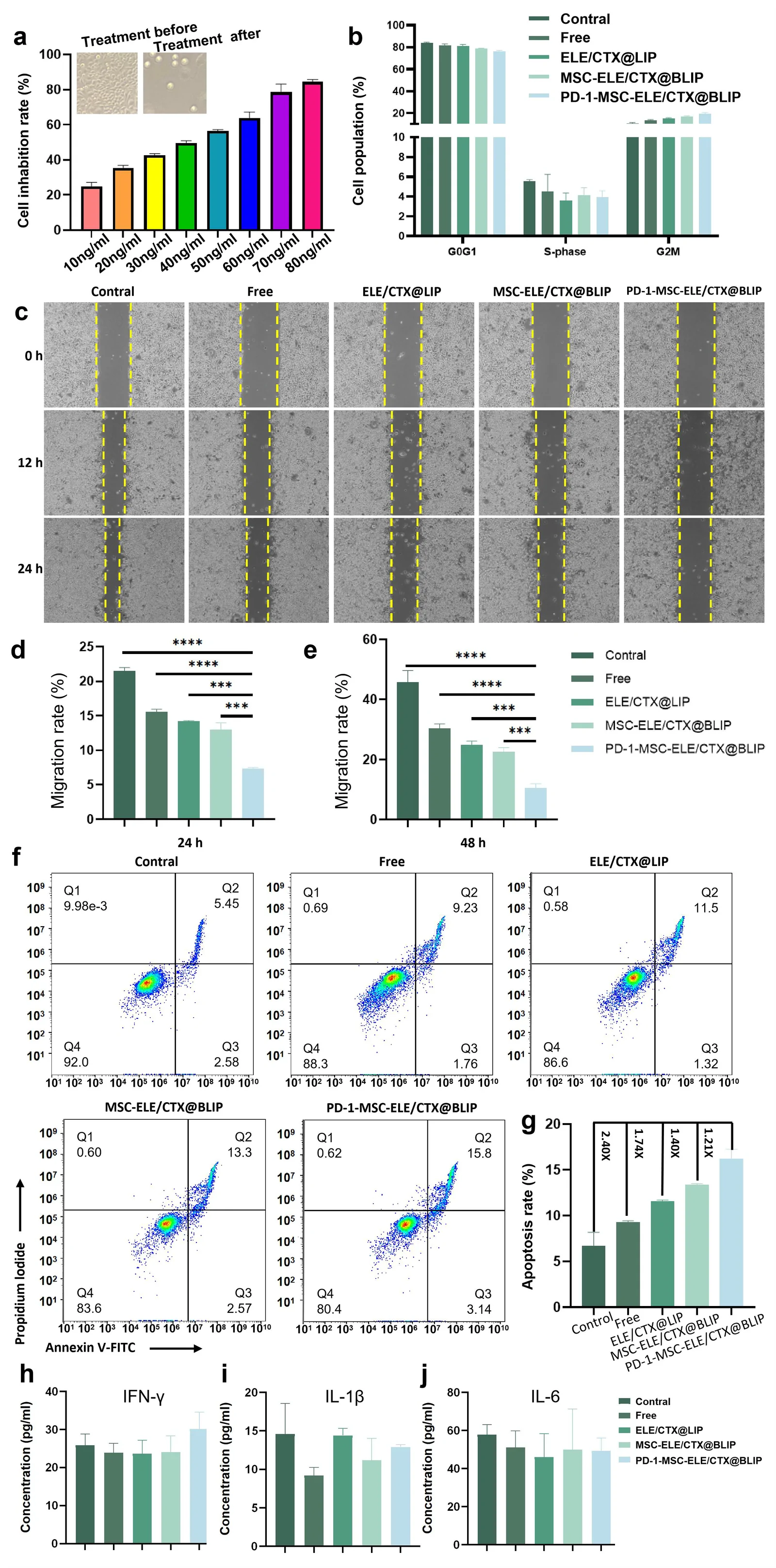
Potentiated antitumor effects of PD-1-MSCs-ELE/CTX@BLIP on G422 cells. **(A)** Concentration-dependent cytotoxicity of PD-1-MSCs-ELE/CTX@BLIP toward G422 cells (n = 3). **(B)** Representative staining images and quantitative analysis of G422 glioma cells after 48 h treatment with the indicated formulations (n = 3). **(C)** Scratch-wound assay illustrating the inhibitory effect of each preparation on G422 cell migration. **(D)** Migration area for 24 h **(E)** Migration area for 48 h **(F)** Representative Annexin V-FITC/PI staining images of G422 cells treated with PD-1-MSCs-ELE/CTX@BLIP for 24 H **(G)** Quantitative assessment of PD-1-MSCs-ELE/CTX@BLIP-induced apoptosis in G422 cells (n = 3). **(H)** ELISA quantification of IFN-γ secretion (n = 3). **(I)** ELISA quantification of IL-1β secretion (n = 3). **(J)** ELISA quantification of IL-6 secretion (n = 3). ***P < 0.001; ****P < 0.0001.

G422 cells were co-incubated with PD-1-MSCs-ELE/CTX@BLIP, MSCs-ELE/CTX@BLIP, ELE/CTX@LIP, or free drugs. The evaluated parameters encompassed cell inhibition rate, cell cycle distribution, apoptosis, and IFN-γ, IL-1β, and IL-6 expression levels. The cytotoxicity of PD-1-MSCs-ELE/CTX@BLIP toward G422 cells increased in a concentration-dependent manner (**Figure 4A**, Supporting Information **Supplementary Figure 5**) (44). Treatment with the various formulations disrupted the cell cycle of G422 cells; compared to the control, PD-1-MSCs-ELE/CTX@BLIP increased the G2/M phase fraction and decreased the S-phase proportion, signifying enhanced induction of apoptosis (**Figure 4B**, Supporting Information **Supplementary Figure 6**). Scratch-wound assays were subsequently performed to evaluate the migratory ability of G422 cells. The findings revealed that nanoparticle encapsulation potentiated the efficacy of the free drugs, and this enhancement was particularly significant following biological modification (**Figures 4C–E**). PD-1-MSCs-ELE/CTX@BLIP inhibited cell migration more effectively than MSCs-ELE/CTX@BLIP, ELE/CTX@LIP, or the free drugs. Apoptosis assays yielded results that are consistent with those of the cell cycle data (**Figure 4F**); PD-1-MSCs-ELE/CTX@BLIP increased apoptosis by 1.21-, 1.40-, 1.74-, and 2.4-fold compared with MSCs-ELE/CTX@BLIP, ELE/CTX@LIP, free drugs, and the control, respectively (**Figure 4G**). The antitumor efficacy of PD-1-MSCs-ELE/CTX@BLIP was further evaluated using ELISA quantification of the tumor-associated factors IFN-γ, IL-1β, and IL-6 secreted directly by G422 cells *in vitro* in the absence of immune cells, as these markers can enhance T cell activity and the antitumor effect in the body. Compared with the control, PD-1-MSCs-ELE/CTX@BLIP significantly increased IFN-γ levels and decreased IL-1β and IL-6 levels, indicating that the treatment alleviated T cell exhaustion and reactivated T cell function, hence augmenting the antitumor response (**Figures 4H–J**) (45). The observed cytokine profile, elevated IFN-γ and reduced IL-6/IL-1β, signifies a microenvironmental shift from immunological suppression to immune activation. The 4.5-fold increase in CD8+ T cell infiltration further supports immunological rejuvenation (46, 47).

### 3.5 *In vivo* targeting of PD-1-MSCs-ELE/CTX@BLIP

**Figure 5:**
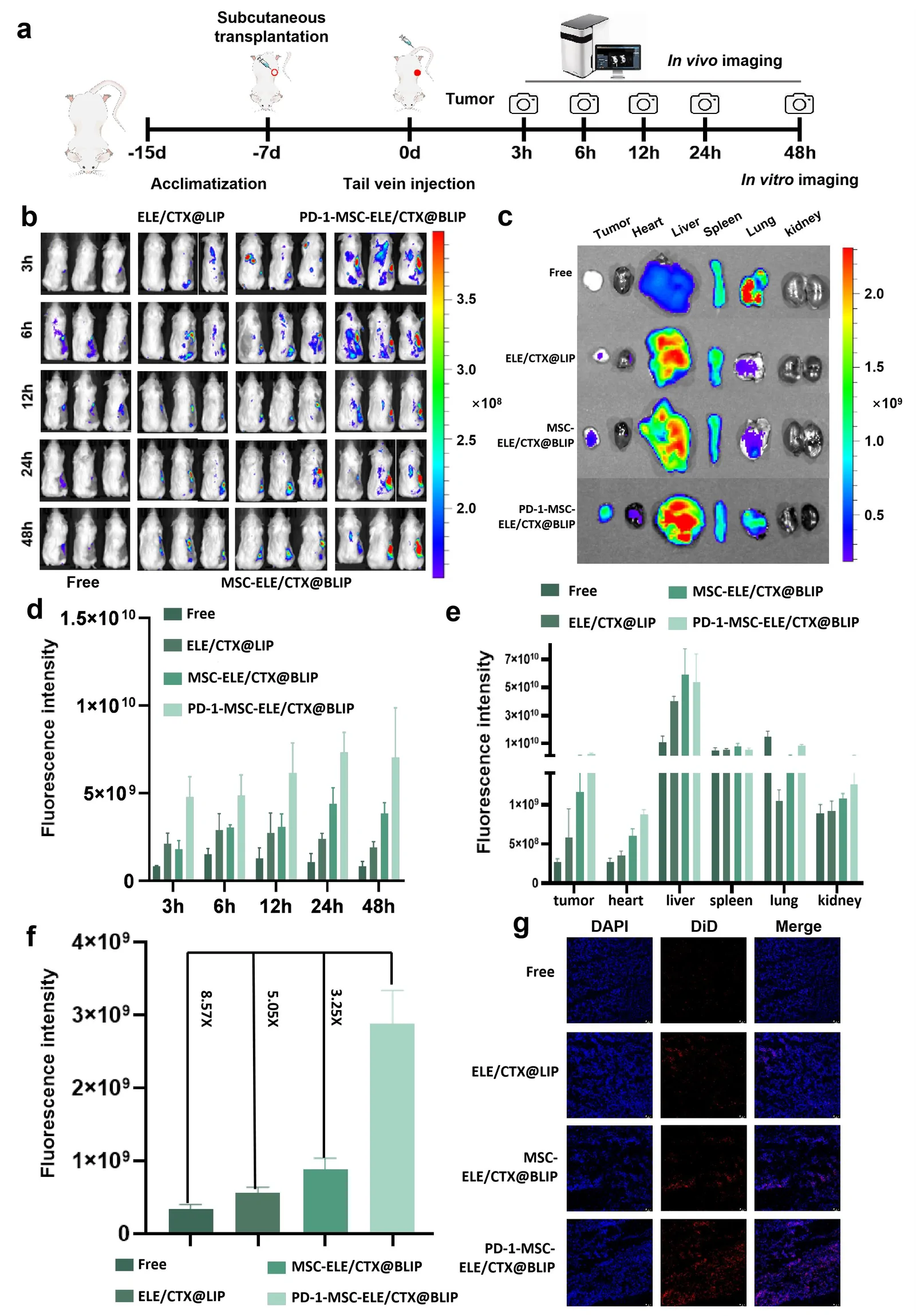
*In vivo* targeting efficacy of PD-1-MSCs-ELE/CTX@BLIP in mice bearing subcutaneous glioma. **(A)** Schematic illustration of the *in vivo* imaging schedule for mice bearing subcutaneous G422 cells. **(B)** Representative fluorescence images for the different formulation groups. **(C)** *Ex vivo* fluorescence images of glioma, brain, heart, liver, spleen, lung, and kidney tissues obtained 48 h post-injection. **(D)** Quantitative analysis of *in vivo* fluorescence in glioma regions at the indicated time points (n = 3). **(E)** Quantitative analysis of *in vitro* fluorescence in glioma regions at the indicated time points (n = 3). **(F)** Quantitative analysis of *in vivo* fluorescence in glioma regions at 48 h (n = 3). **(G)** Fluorescence intensity of glioma tissue sections.

To further evaluate the active targeting capability of PD-1-MSCs-ELE/CTX@BLIP, we created a subcutaneous glioma-bearing model by introducing G422 cells into the right flank of mice (26, 27) and performed *in vivo* fluorescence imaging to determine the distribution (**Figure 5A**). Briefly, PD-1-MSCs-ELE/CTX@BLIP, MSCs-ELE/CTX@BLIP, DiD-loaded ELE/CTX@LIP, and free DiD were intravenously administered through the tail vein to subcutaneous glioma-bearing mice. The free DiD group revealed a weak fluorescent signal, suggesting rapid metabolism or systemic clearance. The ELE/CTX@LIP group attained peak accumulation at 6–12 h, followed by gradual elimination. The MSCs-ELE/CTX@BLIP group peaked at 24 h, with overall accumulation higher than that of the ELE/CTX@LIP group, confirming sustained release and enhanced targeting efficiency (36, 48). Notably, PD-1-MSCs-ELE/CTX@BLIP exhibited significantly higher fluorescence accumulation over the 3–48 h period compared with the other groups, suggesting optimal G422 targeting and prolonged retention (**Figures 5B, D**); this observation holds significant implications for extending the drug’s half-life. *Ex vivo* fluorescence imaging revealed that PD-1-MSCs-ELE/CTX@BLIP was predominantly localized in the tumor and liver, with minimal accumulation in the spleen and lung ((**Figures 5C, E**). Quantitative fluorescence analysis revealed that the fluorescence intensity in the PD-1-MSCs-ELE/CTX@BLIP group was 3.25-, 5.05-, and 8.57-fold higher than that in the MSCs-ELE/CTX@BLIP, ELE/CTX@LIP, and free DiD groups, respectively ((**Figure 5F**), suggesting robust tumor targeting and potential hepatic metabolism, consistent with the quantitative results. Furthermore, the drug concentration in tumors was significantly higher than that in the other treatment groups (Supporting Information **Supplementary Figure 7**). These findings were further confirmed by *ex vivo* fluorescence imaging and histopathological staining ((**Figure 5G**). Notably, liposome accumulation in the liver and kidney could pose a risk of hepatorenal toxicity; therefore, additional studies are warranted to evaluate the safety profile of these liposomes.

### 3.6 *In vivo* antitumor efficacy of PD-1-MSCs-ELE/CTX@BLIP

**Figure 6:**
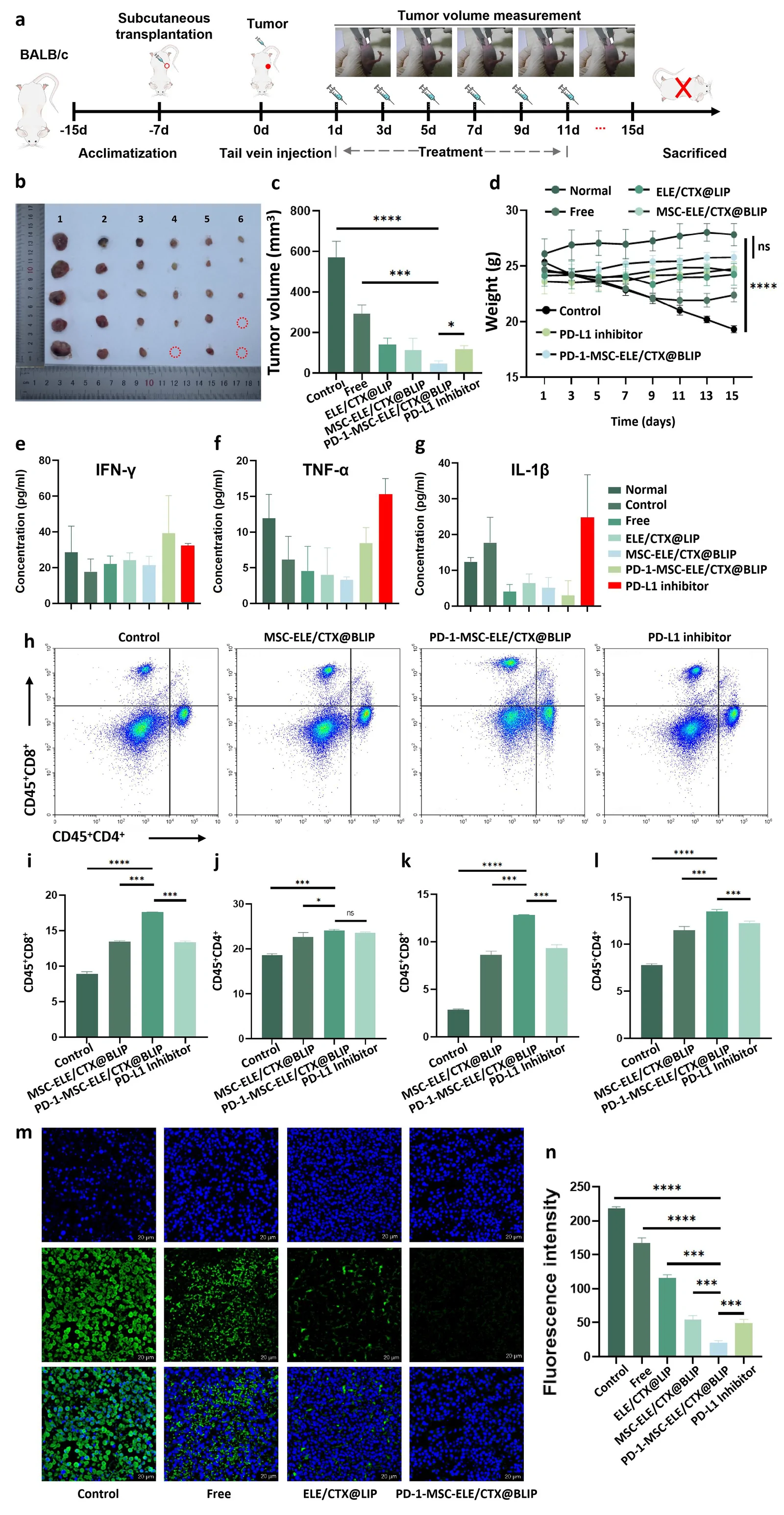
*In vivo* antitumor efficacy of PD-1-MSCs-ELE/CTX@BLIP in BALB/c mice bearing subcutaneous glioma. **(A)** Schematic illustration of the *in vivo* therapeutic schedule for mice bearing subcutaneous G422 cells. **(B)** Photographs of tumor specimens after treatment with (1) Control (2), Free (3), ELE/CTX@LIP (4), MSCs-ELE/CTX@BLIP (5), PD-L1 inhibitor, and (6) PD-1-MSCs-ELE/CTX@BLIP. **(C)** Tumor volume (n = 3). **(D)** Body weight of mice bearing subcutaneous glioma (n = 3). **(E)** ELISA quantification of IFN-γ expression (n = 3). **(F)** ELISA quantification of TNF-α expression (n = 3). **(G)** ELISA quantification of IL-1β expression (n = 3). **(H)** Proportions of CD4+ and CD8+ T cells in the spleen determined by FACS. **(I)** Absolute number of CD8+ T cells in the spleen (n = 3). **(J)** Absolute number of CD4+ T cells in the spleen (n = 3). **(K)** Absolute number of CD8+ T cells in the glioma (n = 3). **(L)** Absolute number of CD4+ T cells in the glioma (n = 3). **(M)** Immunofluorescence staining of Ki-67 in tumor tissues after treatment with the indicated preparations. **(N)** Quantitative immunofluorescence analysis of Ki-67 in tumor tissues. *P < 0.05; ***P < 0.001; ****P < 0.0001; ns, not significant.

We established a subcutaneous glioma mouse model to evaluate the anti-glioma efficacy of PD-1-MSCs-ELE/CTX@BLIP (26, 27). **Figure 6A** depicts the treatment schedule. *In vivo* antitumor efficacy was assessed by monitoring tumor volume and body weight. Compared to the control group, tumor volume in all treatment groups decreased significantly after administration, confirming the clear anti-glioma activity of ELE/CTX. Among all formulations, mice receiving PD-1-MSCs-ELE/CTX@BLIP exhibited the smallest glioma volume (**Figures 6B, C**), signifying a more robust antitumor response; notably, tumors completely disappeared in two mice. Furthermore, body weight did not exhibit a significant decrease in PD-1-MSCs-ELE/CTX@BLIP-treated mice compared with normal mice, demonstrating good biosafety without overt toxicity (**Figure 6D**). To further evaluate the antitumor effect of PD-1-MSCs-ELE/CTX@BLIP, we performed ELISA to quantify tumor-related factors in the TME. IFN-γ, TNF-α, and IL-1β are indicators of T cell activity and antitumor capacity (49). The results revealed that IFN-γ and TNF-α levels in tumors from mice treated with MSCs-ELE/CTX@BLIP, ELE/CTX@LIP, or free drugs were not significantly different from those in the control group. Conversely, PD-1-MSCs-ELE/CTX@BLIP treatment markedly elevated IFN-γ and TNF-α levels and significantly reduced IL-1β, signifying that this regimen alleviated T cell exhaustion and reactivated antitumor T cell activity. These findings further indicate that PD-1-MSCs-ELE/CTX@BLIP specifically targets and binds to PD-L1 on tumor cells, thereby enhancing T cell function, remodeling the TME, and amplifying the antitumor response (**Figures 6E–G**). To determine whether PD-1-MSCs-ELE/CTX@BLIP modulates the immune microenvironment to enhance antitumor efficacy, we analyzed T cell infiltration in the spleen and tumor tissues by flow cytometry (**Figure 6H**, Supporting Information **Supplementary Figure 8**). The spleen, serving as a central hub for lymphocyte activation and recirculation, promoted the delivery of effector cells to tumors through the expansion of CD4+ and CD8+ T cells (**Figures 6I, J**). Compared to the control group, the ratio of tumor-infiltrating CD8+ T cells increased 3.1-fold in the MSCs-ELE/CTX@BLIP group and 4.5-fold in the PD-1-MSCs-ELE/CTX@BLIP group (**Figure 6K**). This 4.5-fold increase in intratumoral CD8+ T cells directly confirms the immune activation effect. Furthermore, tumor uptake increased 5.05-fold (**Figure 5F**). These findings demonstrate a synergistic effect, implying that the nanoplatform helps restore host immunity and eliminate tumor cells (50, 51). The markedly increased infiltration of tumor CD8+ T cells in the PD-1-MSCs-ELE/CTX@BLIP group signifies that MSC membrane modification enhanced drug-loaded nanoparticle accumulation at the tumor site. Furthermore, PD-1 displayed on PD-1-MSCs specifically bound to PD-L1 on tumor cells, thereby relieving T cell inhibition mediated by the PD-1/PD-L1 signaling pathway. Consequently, this enhanced intratumoral CD8+ T cell infiltration reinforced the anti-glioma immune response. These findings revealed that PD-1-MSCs-ELE/CTX@BLIP remodels the TME and potentiates antitumor efficacy. Simultaneously, the proportion of infiltrating CD4+ T cells exhibited an upward trend after treatment (**Figure 6L**), indicating that CD4+ T cells may further augment the CD8+ T cell-mediated antitumor immune response (52). Immunofluorescence and immunohistochemical staining of glioma sections for Ki-67 and GFAP were performed to evaluate tumor cell proliferation and malignancy (53). Immunofluorescence revealed that Ki-67 expression in the PD-1-MSCs-ELE/CTX@BLIP group was significantly lower than that in the experimental and control groups (**Figures 6M, N**), whereas GFAP expression revealed the opposite trend (Supporting Information **Supplementary Figure 9**), suggesting that glioma proliferation and growth were substantially suppressed. Moreover, H&E and immunohistochemical staining jointly confirmed, from a pathological perspective, that PD-1-MSCs-ELE/CTX@BLIP exerted a robust inhibitory effect on glioma growth (Supporting Information **Supplementary Figure 10**).

**Figure 7:**
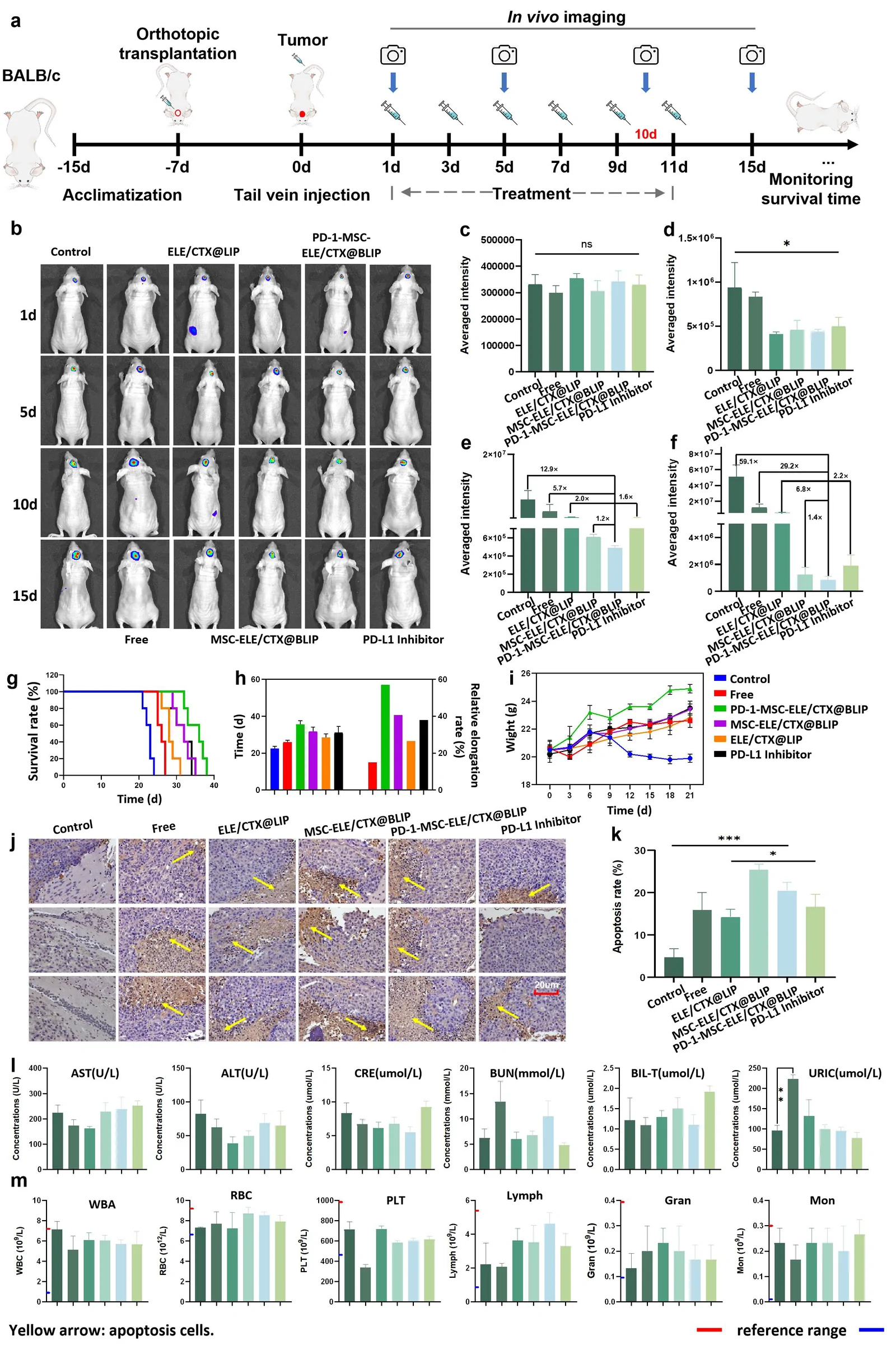
*In vivo* antitumor efficacy of PD-1-MSCs-ELE/CTX@BLIP in BALB/c mice bearing orthotopic glioma. **(A)** Schematic illustration of the *in vivo* therapeutic schedule for mice bearing orthotopic G422-Luc cells. **(B)** Representative fluorescence images for the different formulation groups. **(C)** Average fluorescence intensity of glioma tissues on day 1 after treatment in the different groups (n = 3). **(D)** Average fluorescence intensity of glioma tissues on day 5 after treatment in the different groups (n = 3). **(E)** Average fluorescence intensity of glioma tissues on day 10 post-treatment in the different groups (n = 3). **(F)** Average fluorescence intensity of glioma tissues on day 15 post-treatment in the different groups (n = 3). **(G)** Survival curves for mice bearing orthotopic glioma. **(H)** Survival rate and relative extension of survival time (n = 3). **(I)** Body weight of mice bearing orthotopic glioma (n = 3). **(J)** TUNEL analysis of glioma tissues; arrows indicate apoptotic cells. **(K)** Apoptosis rate of glioma tissues after intervention with different formulations. **(L)** Examination of liver and kidney function indicators after treatment with different formulations (n = 3). **(M)** Blood routine examination after treatment with different formulations (n = 3). *P < 0.05; ***P < 0.001; ns, not significant.

To more directly evaluate the anti-glioma effect of PD-1-MSCs-ELE/CTX@BLIP, we established an orthotopic glioma mouse model (28, 29); **Figure 7A** depicts the treatment schedule. *In vivo* antitumor efficacy was assessed using fluorescence imaging. On day 1 after treatment, no significant difference in fluorescence intensity was observed among any of the treatment groups and the control group (**Figures 7B, C**). After 5 days of treatment, fluorescence intensity in all treated groups was markedly lower than that in the control group, suggesting that the drugs had initiated anti-glioma effects (**Figures 7B, D**). On day 10, the fluorescence signal intensity in the brain tumors of mice treated with PD-1-MSCs-ELE/CTX@BLIP was significantly lower than that in the other groups (**Figure 7B**). Quantitative analysis revealed that the growth-inhibitory effect of PD-1-MSCs-ELE/CTX@BLIP on glioma was 12.9-, 5.7-, 2.0-, 1.2-, and 1.6-fold greater than that of the control, free drug, ELE/CTX@LIP, MSCs-ELE/CTX@BLIP, and PD-L1 inhibitor groups, respectively (**Figure 7E**). Notably, after 15 days of treatment, the fluorescence signal intensity in the brain tumors of mice treated with PD-1-MSCs-ELE/CTX@BLIP exhibited the lowest among all groups (**Figure 7B**). Quantitative analysis revealed that the growth-inhibitory effect of PD-1-MSCs-ELE/CTX@BLIP was 59.1-, 29.2-, 6.8-, 1.4-, and 2.2-fold greater than that of the control, free drug, ELE/CTX@LIP, MSCs-ELE/CTX@BLIP, and PD-L1 inhibitor groups, respectively (**Figure 7F**). These findings revealed that PD-1-MSCs-ELE/CTX@BLIP exerts the most significant inhibitory effect on glioma growth. Median survival time (MST) results revealed that, compared with the control group (22.60 ± 1.14 days), PD-1-MSCs-ELE/CTX@BLIP (35.60 ± 2.07 days), free drug (26.00 ± 1.00 days), ELE/CTX@LIP (28.60 ± 1.82 days), MSCs-ELE/CTX@BLIP (31.80 ± 2.39 days), and PD-L1 inhibitor (31.20 ± 3.35 days) extended survival by 57.52%, 15.04%, 26.55%, 40.71%, and 38.05%, respectively (**Figures 7G, H**). These results signify that ELE/CTX has a clear anti-glioma effect and that encapsulation in PD-1-engineered nanoparticles significantly enhances its efficacy—an outcome attributed to the high BBB-penetrating capacity of MSCs and the augmented, PD-1/PD-L1 axis-mediated targeted drug uptake within glioma lesions (54). The 57.52% extension in survival period indicated that PD-1-engineered nanoparticles may be promising for effective clinical application against glioma (55, 56). These findings were validated through comparison with the PD-L1 inhibitor group. Weight assessment revealed that tumor-bearing mice in the PD-L1 inhibitor, ELE/CTX@LIP, MSCs-ELE/CTX@BLIP, and PD-L1 inhibitor groups exhibited consistent weight gain throughout the treatment period. Conversely, mice in the control group exhibited significant weight gain from day 6 after administration. Mice in the free drug group revealed an initial weight loss followed by gradual recovery starting on day 3. These findings indicate that the drug-loaded nanoparticles suppress glioma growth and improve the overall physical condition of tumor-bearing mice (**Figure 7I**). Collectively, these data affirm the potent anti-glioma efficacy of PD-1-MSCs-ELE/CTX@BLIP. TUNEL immunofluorescence staining of brain tissue sections was employed to assess the ability of PD-1-MSCs-ELE/CTX@BLIP to induce tumor cell apoptosis (57). Compared to the control group, the highest apoptotic rate was observed in mice treated with PD-1-MSCs-ELE/CTX@BLIP and MSCs-ELE/CTX@BLIP, and their pro-apoptotic activity surpassed that of the free drug and ELE/CTX@LIP groups (**Figures 7J, K**).

### 3.7 Biosafety evaluation of PD-1-MSCs-ELE/CTX@BLIP

*In vivo* distribution studies demonstrated that PD-1-MSCs-ELE/CTX@BLIP accumulated predominantly in the liver and spleen. To systematically evaluate biosafety, we performed histological examination, serum biochemistry analyses, and complete blood counts in mice with a tumor. Compared with the normal group, the free drug group exhibited significant neutrophil infiltration in hepatic sinusoids, indicative of hepatotoxicity (Supporting Information **Supplementary Figure 11**) (58). Conversely, no significant histopathological damage was observed in the heart, liver, spleen, lung, or kidney in any nanoparticle-treated group. Serum biochemistry revealed that ALT, AST, total cholesterol, creatinine, and blood urea nitrogen levels were comparable to the control group, whereas uric acid remained within the normal range only in nanoparticle-treated groups, further supporting reduced systemic toxicity (**Figure 7L**). Hematological assessment demonstrated that all parameters, including white blood cells, red blood cells, platelets, lymphocytes, neutrophils, and monocytes, remained within normal physiological ranges (**Figure 7M**, Supporting Information **Supplementary Figure 11**), thereby affirming the lack of hematological toxicity. Collectively, these results indicate that nano-encapsulation significantly attenuates the hepatotoxicity, nephrotoxicity, and hematological toxicity associated with free drugs. Owing to MSC-mediated brain targeting and PD-1-facilitated intratumoral retention, PD-1-MSCs-ELE/CTX@BLIP preferentially concentrates in glioma lesions rather than healthy tissues, thereby reducing off-target exposure and systemic side effects. This advantageous safety profile, combined with its potent anti-glioma efficacy, highlights the promising translational potential of our biomimetic nanoplatform for clinical glioma treatment (59).

## 4 Discussion

Glioma remains the most common and aggressive primary malignant tumor of the central nervous system, with a five-year survival rate below 5% despite substantial advances in multimodal treatment. The BBB and the highly immunosuppressive TME represent two interconnected obstacles that markedly compromise the efficacy of conventional chemotherapy and emerging immunotherapies (60). The BBB prevents over 98% of small-molecule drugs and nearly all biologics from reaching intracranial tumor lesions, whereas glioma cells exploit the PD-1/PD-L1 immune checkpoint pathways to induce CD8+ T cell exhaustion and establish an immunosuppressive microenvironment that resists therapeutic intervention. These dual barriers explain why systemically administered checkpoint inhibitor antibodies, despite their clinical success in peripheral tumors, have yielded disappointing results in glioma clinical trials, largely due to their inability to cross the BBB in sufficient quantities. To overcome these challenges, we developed PD-1-MSCs-ELE/CTX@BLIP, a biomimetic nanoplatform that integrates MSC-mediated BBB penetration, PD-1/PD-L1 axis-mediated active tumor targeting, and concurrent chemoimmunotherapeutic action. We interpret our findings based on existing literature, examine the mechanistic underpinnings of the observed therapeutic efficacy, and address the limitations and translational implications of this strategy.

The core innovation of our nanoplatform is the use of genetically engineered MSC membranes as a biomimetic coating. MSCs possess intrinsic tumor-homing ability and natural BBB traversal capability, making them an attractive vehicle for glioma-targeted drug delivery. Previous studies have demonstrated that MSC membrane-coated nanoparticles exhibit enhanced targeted recognition and cellular uptake by glioblastoma cells compared with uncoated nanoparticles. However, the heterogeneity of primary MSCs and the limited availability of scalable cell sources have constrained the translational potential of this approach (15, 61). To address this limitation, we established a stable PD-1-overexpressing MSC line through lentiviral transduction, providing a stable and scalable source of functionalized membranes. Membrane localization of PD-1 was confirmed by co-localization with GFP-tagged PD-1 and further verified at the transcriptional and translational protein levels, ensuring that the surface-displayed immune checkpoint molecule would be available for targeting and immunomodulatory functions.

Physicochemical characterization demonstrated that PD-1-MSCs-ELE/CTX@BLIP revealed favorable attributes for *in vivo* application. The mean particle size, PDI, and ζ-potential are consistent with those previously reported cell membrane-coated nanoparticles that exhibit prolonged circulation and enhanced tumor accumulation (62). Encapsulation efficiencies exceeding 90% for ELE and CTX, together with excellent storage stability over 15 days at 4 °C, highlight the robustness of the formulation. The negligible hemotoxicity (< 5%) further confirmed the biocompatibility of the PD-1-MSC membrane coating, which is essential for intravenous administration. Moreover, SDS-PAGE analysis revealed that the protein profile of PD-1-MSCs-ELE/CTX@BLIP closely resembled that of PD-1-MSCsM, with a distinct PD-1 band distinguishing it from non-engineered MSCs-ELE/CTX@BLIP. This protein preservation is essential because membrane-associated proteins, including adhesion molecules, homing receptors, and immune checkpoint molecules, mediate the biological functions of biomimetic nanoparticles (63). TEM and confocal microscopy further confirmed the core-shell architecture, with the liposomal core uniformly enveloped by the PD-1-MSC membrane shell, a configuration that maximizes the functional benefits of both components.

One of the findings of this study is the markedly enhanced BBB penetration and glioma-targeting capability conferred by PD-1 functionalization. In the *in vitro* BBB model established by bEnd.3 monolayers, PD-1-MSCs-ELE/CTX@BLIP exhibited significantly higher DiD fluorescence intensity and drug content in the lower chamber compared with ELE/CTX@LIP and MSCs-ELE/CTX@BLIP. This observation aligns with the established role of MSC membranes to facilitate transendothelial migration. However, the additional enhancement beyond that of non-engineered MSC membranes indicates that PD-1 surface display may contribute to BBB traversal beyond simple membrane biomimicry (64). Although the underlying mechanism requires further investigation, we hypothesize that PD-1 may interact with low-level PD-L1 expressed on activated brain endothelial cells under inflammatory conditions, thereby facilitating active transcytosis.

The cellular targeting studies further demonstrated that the PD-1/PD-L1 axis is the driver of enhanced glioma accumulation. PD-1-MSCs-ELE/CTX@BLIP exhibited 6.26-fold higher uptake by G422 cells compared with free DiD, and this benefit was completely abrogated by PD-L1 inhibitor pretreatment, reducing uptake to levels comparable to those of MSCs-ELE/CTX@BLIP. This PD-1/PD-L1-dependent selectivity resembles the mechanism of anti-PD-L1 antibody-functionalized nanocarriers recently reported for glioblastoma targeting but offers the distinct advantage of incorporating the checkpoint-blocking function directly into the carrier membrane rather than requiring additional antibody conjugation (37). Furthermore, the absence of enhanced uptake in PD-L1-negative 4T1 cells confirmed the specificity of this interaction, supporting the safety profile of the nanoplatform by sparing PD-L1-negative normal tissues.

The *in vivo* fluorescence imaging results further confirmed the active targeting capability. PD-1-MSCs-ELE/CTX@BLIP reached maximal tumor accumulation at 24 h post-injection and maintained significantly greater fluorescence intensity than all control formulations throughout the 48 h observation period. This superior tumor accumulation can be attributed to the dual targeting mechanism: MSC membrane-mediated homing to glioma lesions, followed by PD-1/PD-L1-mediated retention within the tumor microenvironment. This “homing-plus-retention” strategy addresses a critical limitation of conventional MSC membrane-coated nanoparticles, which, although capable of crossing the BBB and reaching glioma lesions, frequently exhibit inadequate intratumoral retention (65). The prolonged retention observed in our study is particularly noteworthy because most intravenously administered nanoparticles undergo rapid clearance from tumors within 24 h.

The therapeutic efficacy of PD-1-MSCs-ELE/CTX@BLIP was assessed across multiple dimensions. The concentration-dependent cytotoxicity against G422 cells, with enhanced G2/M phase arrest and apoptosis induction, reflects the synergistic actions of ELE and CTX. Cabazitaxel, as a second-generation taxane, exerts potent anti-proliferative effects by stabilizing microtubules and arresting cells in the G2/M phase. Elemene, a naturally occurring lipophilic sesquiterpene isolated from *Curcuma wenyujin*, exerts anti-glioma activity through multiple mechanisms, including apoptosis induction, reversal of multidrug resistance, and immunomodulation. The synergistic combination of these two agents within a single nanocarrier likely contributes to the enhanced efficacy observed compared with either agent alone (9).

Beyond its chemotherapeutic effects, PD-1-MSCs-ELE/CTX@BLIP demonstrated pronounced immunomodulatory activity. In the subcutaneous glioma model, treatment with PD-1-MSCs-ELE/CTX@BLIP markedly elevated intratumoral IFN-γ and TNF-α levels and significantly reduced IL-1β, suggesting a transition from immune suppression to immune activation. This cytokine profile is consistent with relief of T cell exhaustion and reactivation of effector T cell function. Flow cytometry analysis further revealed that the proportion of tumor-infiltrating CD8+ T cells increased 4.5-fold in the PD-1-MSCs-ELE/CTX@BLIP group compared with the control, significantly higher than the 3.1-fold increase observed with MSCs-ELE/CTX@BLIP. This enhanced CD8+ T cell infiltration is likely attributable to the PD-1/PD-L1 checkpoint blockade function of the nanoplatform: surface-displayed PD-1 competitively binds to PD-L1 on glioma cells and tumor-associated macrophages, preventing the engagement of PD-1 on T cells and thereby rescuing them from exhaustion (66). Recent studies have demonstrated that glioma cells produce soluble PD-L1 through the Wnt/β-catenin signaling pathway, which interacts with PD-1 on CD8+ T cells and suppresses their function by reducing IFN-γ levels. By intercepting this PD-L1-mediated suppression, PD-1-MSCs-ELE/CTX@BLIP effectively restores CD8^+^ T cell activity (67).

The orthotopic glioma model provided the most clinically relevant evaluation of therapeutic efficacy. PD-1-MSCs-ELE/CTX@BLIP treatment produced the most pronounced inhibition of intracranial tumor growth and extended the median survival time by 57.52%. This survival benefit compares favorably with recently developed glioblastoma nanotherapeutic strategies. For instance, anti-PD-L1 scFv-functionalized lipid nanocarriers loaded with temozolomide demonstrated significant survival improvement in the GL261/C57BL/6 model, whereas biomimetic nanoparticles based on immortalized MSCs loaded with shCD73 and doxorubicin revealed therapeutic efficacy in delaying glioma progression (68). The 57.52% survival extension achieved in our study is particularly encouraging because it was attained without the addition of exogenous immune checkpoint antibodies, relying instead on the intrinsic PD-1 displayed on the nanoparticle surface. This self-contained checkpoint blockade strategy simplifies the formulation and potentially reduces the immunogenicity and cost associated with antibody-based therapies.

To further contextualize our findings, PD-1-MSCs-ELE/CTX@BLIP was compared with other recently developed glioma-targeted biomimetic nanoplatforms. Cancer cell membrane-coated nanoparticles provide homotypic targeting advantages, which are susceptible to macrophage-mediated clearance and may elicit unwanted immune responses because of the presentation of tumor-associated antigens. MSC membrane-coated nanoparticles address this limitation by providing immune-evasive and tumor-homing properties without the immunogenicity concerns of tumor cell membranes (10). However, non-engineered MSC membranes lack the molecular specificity required for robust intratumoral retention. Our PD-1-functionalized approach bridges this gap by adding a second layer of targeting specificity. Compared with antibody-functionalized nanoparticles, this approach integrates the targeting ligand (PD-1) directly into the membrane protein repertoire, eliminating the need for additional conjugation chemistry that may compromise nanoparticle stability or biological activity (69). Moreover, the dual functionality of PD-1, serving simultaneously as a targeting ligand and an immune checkpoint-blocking agent, represents a conceptual advance over platforms that separate these functions into distinct components.

The selection of ELE and CTX as the therapeutic payload further distinguishes this nanoplatform. Although temozolomide remains the standard chemotherapeutic agent for glioblastoma, its clinical efficacy is limited by acquired drug resistance and suboptimal BBB penetration. CTX, a second-generation taxane, has demonstrated promising preclinical efficacy in glioblastoma models, and its combination with ELE, a traditional Chinese medicine-derived agent with established clinical safety and immunomodulatory properties, offers a rational strategy for concurrent chemoimmunotherapy. The 98.5% and 94.2% encapsulation efficiencies for ELE and CTX, respectively, ensure that the synergistic drug ratio is maintained upon systemic administration, an essential factor for combination therapy (9).

The biosafety evaluation demonstrated that PD-1-MSCs-ELE/CTX@BLIP exhibited a favorable safety profile. Unlike the free drug group, nanoparticle-treated groups revealed no significant histopathological abnormalities in major organs. Additionally, serum biochemistry indices and hematological parameters remained within normal physiological ranges in all nanoparticle-treated groups. This reduced systemic toxicity is likely attributable to several complementary factors (1): The MSC membrane coating provides immune evasion and prolongs systemic circulation, thereby reducing off-target accumulation (2); PD-1-mediated active targeting preferentially concentrates the therapeutic payload within the tumor tissues, and minimizes exposure to healthy tissues; and (3) the liposomal encapsulation of ELE and CTX reduces the peak plasma concentrations of free drugs, mitigating dose-dependent toxicities (70, 71). The preferential accumulation of PD-1-MSCs-ELE/CTX@BLIP in tumor tissues over healthy organs, as demonstrated by *ex vivo* fluorescence imaging, further supports the safety advantage of this targeted delivery strategy.

However, several limitations of this study should be addressed. First, although the G422/BALB/c model provides valuable proof-of-concept data, the translational relevance to human glioma requires confirmation in patient-derived xenograft models or immunocompetent models with humanized immune systems. The immunological landscape of murine glioma models differs substantially from that of human glioblastoma, particularly regarding the composition and functionality of tumor-infiltrating lymphocytes. Second, the long-term *in vivo* fate of PD-1-MSCs-ELE/CTX@BLIP—including its biodegradation, clearance pathways, and potential chronic toxicity—remains to be comprehensively characterized. Although our 15-day stability data and subacute toxicity assessment are encouraging, extended observation periods are necessary to exclude delayed adverse effects. Third, the detailed molecular mechanisms underlying the interaction between nanoparticle-surface PD-1 and PD-L1, and the downstream signaling pathways involved in TME remodeling, warrant deeper investigation. Whether PD-1-MSCs-ELE/CTX@BLIP modulates additional immune checkpoint pathways or influences other immunosuppressive cell populations, including myeloid-derived suppressor cells or tumor-associated macrophages, remains unknown. Fourth, the scalability and manufacturing reproducibility of PD-1-MSC membrane-coated nanoparticles present important challenges for clinical translation. Specifically, the lentiviral transduction and membrane isolation processes require further optimization for large-scale production under good manufacturing practice conditions.

Building upon the promising findings of this study, several future research directions warrant exploration. First, optimizing the PD-1 expression level on the MSC membrane may enhance tumor-targeting efficiency and immune checkpoint-blocking potency without compromising membrane integrity. Second, incorporating alternative therapeutic payloads—including immunostimulatory agents, siRNA, or mRNA—could expand the applicability of this platform beyond chemotherapy. Third, combining PD-1-MSCs-ELE/CTX@BLIP with complementary therapeutic modalities, including radiotherapy or focused ultrasound-mediated BBB opening, may further improve therapeutic synergy. Fourth, the development of a humanized version of this platform incorporating human MSC membranes and human PD-1 would facilitate clinical translation. Furthermore, the potential of this biomimetic strategy for treating other PD-L1-positive intracranial malignancies, including brain metastases, warrants further investigation.

## 5 Conclusion

We developed a PD-1-functionalized MSC membrane-camouflaged biomimetic nanoplatform co-loaded with ELE and CTX for glioma treatment. This nanosystem exhibited favorable physicochemical characteristics, excellent biocompatibility, and strong ability (6.26-fold) to cross the blood-brain barrier. Through the PD-1/PD-L1 recognition axis, it achieves active targeting and high accumulation in glioma tissues (8.57-fold). The nanoplatform directly kills glioma cells, inhibits tumor proliferation, blocks immune checkpoint signaling, relieves T-cell exhaustion, and remodels the immunosuppressive tumor microenvironment. In an orthotopic glioma model, it significantly suppresses tumor growth and prolongs the survival (57.52%) of tumor-bearing mice without overt systemic toxicity. Collectively, these findings demonstrate that this biomimetic nanodelivery system integrates targeted chemotherapy with immune activation, offering a promising and translatable strategy for precision glioma therapy.

## Data Availability

The original contributions presented in the study are included in the article/**Supplementary Material**. Further inquiries can be directed to the corresponding authors.
