# Supplementary material for: Biomimetic PD-1-MSCs membrane-engineered nanoparticles for enhanced blood-brain barrier penetration and anti- glioma therapy
Source: Front Immunol. 2026 Aug 18;17:1906527. doi: 10.3389/fimmu.2026.1906527 (PMC13529769; doi:10.3389/fimmu.2026.1906527)
Supplement: Supplementary file 1 [file Table1.docx]

**Table S1 Information related to the PD-1 plasmid.**

| **NO.** | H28933 | **Gene** | Pdcd1 |
| --- | --- | --- | --- |
| **GenBank ID** | NM_008798.3 | **Size of the gene** | 867bp |
| **Species** | Mouse | **Restriction site** | EcoRI |
| **Resistance** | Amp | | |
| **Vector** | GL107 pSLenti-EF1-EGFP-P2A-Puro-CMV-MCS-3xFLAG-WPRE | | |
| **Plasmid** | pSLenti-EF1-EGFP-P2A-Puro-CMV-Pdcd1-3xFLAG-WPRE | | |
| **For primer** | CMV-F CGCAAATGGGCGGTAGGCGTG | | |
| **Rev primer** | 101-R AGAGACAGCAACCAGGAT | | |

**Table S2 Particle sizes, PDI, ζ-potentials of the drug in 3 liposomes.**

| **Samples** | **Size/nm** | **PDI** | **ζ- poteneial/mV** | **EE (%)** | |
| --- | --- | --- | --- | --- | --- |
|  |  |  |  | **ELE** | **CTX** |
| PD-1-MSCs-ELE/CTX@BLIP | 135.9±4.6 | 0.182±0.043 | -11.72±0.25 | 98.45 | 94.17 |
| MSCs-ELE/CTX@BLIP | 149.4±9.1 | 0.207±0.017 | -9.80±0.05 | 98.60 | 94.51 |
| ELE/CTX@LIP | 112.3±1.1 | 0.195±0.065 | -8.70±0.15 | 97.43 | 97.74 |

**FIGURE S1 PD-1 plasmid schematic diagram.**

**FIGURE S2 The construction result of functionalized PD-1-MSCs.**

**FIGURE S3 Particle size, PDI and ζ-potential stability for 15 days (n=3).**

**FIGURE S4 TEM image of nanoparticles. (a, b**) **Liposomes of different multiples. (c**) **MSC-ELE/CTX@BLIP. (d**) **PD-1-MSC-ELE/CTX@BLIP.**

**FIGURE S5 The inhibitory effect of PD-1-MSC-ELE/CTX@BLIP on G422 glioma cells.**

**FIGURE S6 Representative results of staining and quantitative analysis of G422 glioma cells after being treated with different preparations for 48 h.**

**FIGURE S7 Quantitative analysis of in vivo bioluminescence in glioma regions at different time points (n=3).**

**FIGURE S8 The proportion of CD4+/CD8+ T cells in the tumor microenvironment was determined by FACS.**

**FIGURE S9 Immunofluorescence staining was performed on the tumor tissues that had undergone different treatments to detect the expression of GFAP and ki-67 in these tissues.**

**FIGURE S10 H&E staining and immunohistochemical evaluation was conducted to assess the expression of GFAP and Ki-67 in tumor tissues after being treated with different preparations.**

**FIGURE S11 Biological safety evaluation of PD-1-MSCs-ELE/CTX@BLIP (n=3).**
